# Extensive loss of cell-cycle and DNA repair genes in an ancient lineage of bipolar budding yeasts

**DOI:** 10.1371/journal.pbio.3000255

**Published:** 2019-05-21

**Authors:** Jacob L. Steenwyk, Dana A. Opulente, Jacek Kominek, Xing-Xing Shen, Xiaofan Zhou, Abigail L. Labella, Noah P. Bradley, Brandt F. Eichman, Neža Čadež, Diego Libkind, Jeremy DeVirgilio, Amanda Beth Hulfachor, Cletus P. Kurtzman, Chris Todd Hittinger, Antonis Rokas

**Affiliations:** 1 Department of Biological Sciences, Vanderbilt University, Nashville, Tennessee, United States of America; 2 Laboratory of Genetics, Genome Center of Wisconsin, DOE Great Lakes Bioenergy Research Center, Wisconsin Energy Institute, J. F. Crow Institute for the Study of Evolution, University of Wisconsin–Madison, Wisconsin, United States of America; 3 Guangdong Province Key Laboratory of Microbial Signals and Disease Control, Integrative Microbiology Research Centre, South China Agricultural University, Guangzhou, China; 4 University of Ljubljana Biotechnical Faculty, Department of Food Science and Technology, University of Ljubljana, Ljubljana, Slovenia; 5 Laboratorio de Microbiología Aplicada, Biotecnología y Bioinformática, Instituto Andino Patagónico de Tecnologías Biológicas y Geoambientales, Universidad Nacional del Comahue-CONICET, San Carlos de Bariloche, Río Negro, Argentina; 6 Mycotoxin Prevention and Applied Microbiology Research Unit, National Center for Agricultural Utilization Research, Agricultural Research Service, United States Department of Agriculture, Peoria, Illinois, United States of America; 7 Laboratory of Genetics, Genome Center of Wisconsin, Wisconsin Energy Institute, J. F. Crow Institute for the Study of Evolution, University of Wisconsin–Madison, Madison, Wisconsin, United States of America; The Sainsbury Laboratory, UNITED KINGDOM

## Abstract

Cell-cycle checkpoints and DNA repair processes protect organisms from potentially lethal mutational damage. Compared to other budding yeasts in the subphylum Saccharomycotina, we noticed that a lineage in the genus *Hanseniaspora* exhibited very high evolutionary rates, low Guanine–Cytosine (GC) content, small genome sizes, and lower gene numbers. To better understand *Hanseniaspora* evolution, we analyzed 25 genomes, including 11 newly sequenced, representing 18/21 known species in the genus. Our phylogenomic analyses identify two *Hanseniaspora* lineages, a faster-evolving lineage (FEL), which began diversifying approximately 87 million years ago (mya), and a slower-evolving lineage (SEL), which began diversifying approximately 54 mya. Remarkably, both lineages lost genes associated with the cell cycle and genome integrity, but these losses were greater in the FEL. E.g., all species lost the cell-cycle regulator WHIskey 5 (*WHI5*), and the FEL lost components of the spindle checkpoint pathway (e.g., Mitotic Arrest-Deficient 1 [*MAD1*], Mitotic Arrest-Deficient 2 [*MAD2*]) and DNA-damage–checkpoint pathway (e.g., Mitosis Entry Checkpoint 3 [*MEC3*], RADiation sensitive 9 [*RAD9*]). Similarly, both lineages lost genes involved in DNA repair pathways, including the DNA glycosylase gene 3-MethylAdenine DNA Glycosylase 1 (*MAG1*), which is part of the base-excision repair pathway, and the DNA photolyase gene PHotoreactivation Repair deficient 1 (*PHR1*), which is involved in pyrimidine dimer repair. Strikingly, the FEL lost 33 additional genes, including polymerases (i.e., POLymerase 4 [*POL4*] and *POL32*) and telomere-associated genes (e.g., Repressor/activator site binding protein-Interacting Factor 1 [*RIF1*], Replication Factor A 3 [*RFA3*], Cell Division Cycle 13 [*CDC13*], Pbp1p Binding Protein [*PBP2*]). Echoing these losses, molecular evolutionary analyses reveal that, compared to the SEL, the FEL stem lineage underwent a burst of accelerated evolution, which resulted in greater mutational loads, homopolymer instabilities, and higher fractions of mutations associated with the common endogenously damaged base, 8-oxoguanine. We conclude that *Hanseniaspora* is an ancient lineage that has diversified and thrived, despite lacking many otherwise highly conserved cell-cycle and genome integrity genes and pathways, and may represent a novel, to our knowledge, system for studying cellular life without them.

## Abstract 2

A lineage in the budding yeast genus Hanseniaspora has remarkably high evolutionary rates, low GC content, small genome sizes, and lower gene numbers than the rest of the subphylum. Analysis of 25 genomes reveals that this lineage has survived and diversified for tens of millions of years despite lacking dozens of cell cycle and DNA repair genes normally thought to be essential.

## Introduction

Genome maintenance is largely attributed to the fidelity of cell-cycle checkpoints, DNA repair pathways, and their interaction [1]. Dysregulation of these processes often leads to the loss of genomic integrity [2] and hypermutation or the acceleration of mutation rates [3]. E.g., improper control of cell-cycle and DNA repair processes can lead to 10- to 100-fold increases in mutation rate [4]. Furthermore, deletions of single genes can have profound effects on genome stability. E.g., the deletion of Mitosis Entry Checkpoint 3 (*MEC3*), which is involved in sensing DNA damage in the G1 and G2/M cell-cycle phases, can lead to a 54-fold increase in the gross chromosomal rearrangement rate [5]. Similarly, nonsense mutations in mismatch repair proteins account for the emergence of hypermutator strains in the yeast pathogens *Cryptococcus deuterogattii* [6] and *C*. *neoformans* [7,8]. Because of their importance in ensuring genomic integrity, most genome-maintenance–associated processes are thought to be evolutionarily ancient and broadly conserved [9].

One such ancient and highly conserved process in eukaryotes is the cell cycle [10,11]. Landmark features of cell-cycle control include cell-size control, the mitotic spindle checkpoint, the DNA-damage–response checkpoint, and DNA replication [9]. Cell size is controlled, in part, through the activity of WHIskey 5 (*WHI5*), which represses the G1/S transition by inhibiting G1/S transcription [12]. Similarly, when kinetochores are improperly attached or are not attached to microtubules, the mitotic spindle checkpoint helps to prevent activation of the Anaphase-Promoting Complex (APC), which controls the G1/S and G2/M transitions [9,13]. Additional key regulators in this process are Mitotic Arrest-Deficient 1 (Mad1) and Mad2, which dimerize at unattached kinetochores and delay anaphase. Failure of Mad1:Mad2 recruitment to unattached kinetochores results in failed checkpoint activity [14]. Importantly, many regulators, including but not limited to those mentioned here, are highly similar in structure and function between fungi and animals and are thought to have a shared ancestry [10]. Interestingly, cell-cycle initiation in certain fungi (including *Hanseniaspora*) is achieved through SWItching deficient (*SWI*) 4/6 cell-cycle box-binding factor (SBF), a transcription factor that is functionally equivalent but evolutionarily unrelated to E2 promoter binding Factor (E2F), the transcription factor that that initiates the cycle in animals, plants, and certain early-diverging fungal lineages [11]. SBF is postulated to have been acquired via a viral infection, suggesting that evolutionary changes in this otherwise highly conserved process can and do rarely occur [11,15].

DNA damage checkpoints can arrest the cell cycle and influence the activation of DNA repair pathways, the recruitment of DNA repair proteins to damaged sites, and the composition and length of telomeres [16]. E.g., *MEC3* and RADiation sensitive 9 (*RAD9*) function as checkpoint genes required for arrest in the G2 phase after DNA damage has occurred [17]. Additionally, the deletions of DNA damage and checkpoint genes have been known to cause hypermutator phenotypes in the baker’s yeast *Saccharomyces cerevisiae* [18]. Similarly, hypermutator phenotypes are associated with loss-of-function mutations in DNA polymerase genes [19]. E.g., deletion of the DNA polymerase δ subunit gene, POLymerase 32 (*POL32*), which participates in multiple DNA repair processes, causes an increased mutational load and hypermutation in *S*. *cerevisiae*, in part through the increase of genomic deletions and small indels [18,20]. Likewise, the deletion of 3-MethylAdenine DNA Glycosylase 1 (*MAG1*), a gene encoding a DNA glycosylase that removes damaged bases via the multistep base-excision repair pathway, can cause a 2,500-fold increased sensitivity to the DNA alkylating agent methyl methanesulfonate [21].

In contrast to genes in multistep DNA repair pathways, other DNA repair genes function individually or are parts of simpler regulatory processes. E.g., PHotoreactivation Repair-deficient 1 (*PHR1*), a gene that encodes a photolyase, is activated in response to and repairs pyrimidine dimers, one of the most frequent types of lesions caused by damaging UV light [22,23]. Other DNA repair genes do not interact with DNA but function to prevent the misincorporation of damaged bases. E.g., Peroxisomal Coenzyme A Diphosphatase 1 (*PCD1*) encodes a 8-oxo-dGTP diphosphatase [24], which suppresses G → T or C → A transversions by removing 8-oxo-dGTP, thereby preventing the incorporation of the base 8-oxo-dG, one of the most abundant endogenous forms of an oxidatively damaged base [24–26]. Collectively, these studies demonstrate that the loss of DNA repair genes can lead to hypermutation and increased sensitivity to DNA-damaging agents.

Hypermutation phenotypes are generally short-lived because most mutations are deleterious and are generally adaptive only in highly stressful or rapidly fluctuating environments [27]. E.g., in *Pseudomonas aeruginosa* infections of cystic fibrosis patients [28] and mouse-gut–colonizing *Escherichia coli* [29], hypermutation is thought to facilitate adaptation to the host environment and the evolution of drug resistance. Similarly, in the fungal pathogens *C*. *deuterogattii* [6], *C*. *neoformans* [7,8], and *Candida glabrata* [30], hypermutation is thought to contribute to within-host adaptation, which may involve modulating traits such as drug resistance [6,30]. However, as adaptation to a new environment increases, hypermutator alleles are expected to decrease in frequency because of the accumulation of deleterious mutations that result as a consequence of the high mutation rate [31,32]. In agreement with this prediction, half of the experimentally evolved hypermutating lines of *S*. *cerevisiae* had reduced mutation rates after a few thousand generations [33], suggesting hypermutation is a short-lived phenotype and that compensatory mutations can restore or lower the mutation rate. Additionally, this experiment also provided insights to how strains may cope with hypermutation; e.g., all *S*. *cerevisiae* hypermutating lines increased their ploidy, presumably to reduce the impact of higher mutation rates [33]. Altogether, hypermutation can produce short-term advantages but causes long-term disadvantages, which may explain its repeated but short-term occurrence in clinical environments [29] and its sparseness in natural ones. While these theoretical and experimental studies have provided seminal insights into the evolution of mutation rates and hypermutation, we still lack understanding of the long-term, macroevolutionary effects of increased mutation rates.

Recently, multiple genome-scale phylogenies of species in the budding yeast subphylum Saccharomycotina showed that certain species in the bipolar budding yeast genus *Hanseniaspora* are characterized by very long branches [34–36], which are reminiscent of the very long branches of fungal hypermutator strains [6–8]. Most of what is known about these cosmopolitan yeasts relates to their high abundance on mature fruits and in fermented beverages [37], especially on grapes and in wine must [38,39]. As a result, *Hanseniaspora* plays a significant role in the early stages of fermentation and can modify wine color and flavor through the production of enzymes and aroma compounds [40]. Surprisingly, even with the use of *S*. *cerevisiae* starter cultures, *Hanseniaspora* species, particularly *Hanseniaspora uvarum*, can achieve very high cell densities, in certain cases comprising greater than 80% of the total yeast population, during early stages of fermentation [41], suggesting exceptional growth capabilities in this environment.

To gain insight into the long branches and the observed fast growth of *Hanseniaspora*, we sequenced and extensively characterized gene content and patterns of evolution in 25 genomes, including 11 newly sequenced for this study, from 18/21 known species in the genus. Our analyses showed that species in the genus *Hanseniaspora* lost many genes involved in diverse processes and delineated two lineages within the genus: a faster-evolving lineage (FEL), which has a strong signature of acceleration in evolutionary rate at its stem branch and has lost many additional genes involved in diverse processes, and a slower-evolving lineage (SEL), which has a weaker signature of evolutionary rate acceleration at its stem branch and underwent fewer gene losses. Specifically, compared to *S*. *cerevisiae*, there are 748 genes that were lost from two-thirds of *Hanseniaspora* genomes, with FEL yeasts having lost an additional 661 genes and SEL yeasts having lost only an additional 23. Relaxed molecular clock analyses estimate that the FEL and SEL split approximately 95 million years ago (mya). The degree of evolutionary rate acceleration is commensurate with the preponderance of loss of genes associated with cell-cycle and DNA repair processes. Both lineages have lost major cell-cycle regulators, including *WHI5* and components of the APC, while FEL species additionally lost numerous genes associated with the spindle checkpoint (e.g., *MAD1* and *MAD2*) and the DNA damage checkpoint (e.g., *MEC3* and *RAD9*). Similar patterns are observed among DNA-repair–related genes: *Hanseniaspora* species have lost 14 genes, while the FEL yeasts have lost an additional 33 genes. E.g., both lineages have lost *MAG1* and *PHR1*, while the FEL has lost additional genes, including polymerases (i.e., *POL32* and *POL4*) and multiple telomere-associated genes (e.g., Repressor/activator site binding protein-Interacting Factor 1 [*RIF1*], Replication Factor A 3 [*RFA3*], Cell Division Cycle 13 [*CDC13*], Pbp1p Binding Protein 2 [*PBP2*]). Compared to the SEL, analyses of substitution patterns in the FEL show higher levels of sequence substitutions, greater instability of homopolymers, and a greater mutational signature associated with the commonly damaged base, 8-oxo-dG [26]. Furthermore, we find that the transition to transversion (or transition/transversion) ratios of the FEL and the SEL are both very close to the ratio expected if transitions and transversions occur neutrally. These results are consistent with the hypothesis that species in the FEL represent a novel, to our knowledge, example of diversification and long-term evolutionary survival of a hypermutator lineage, which highlights the potential of *Hanseniaspora* for understanding the long-term effects of hypermutation on genome function and evolution.

## Results

### An exceptionally high evolutionary rate in the FEL stem branch

**Fig 1:**
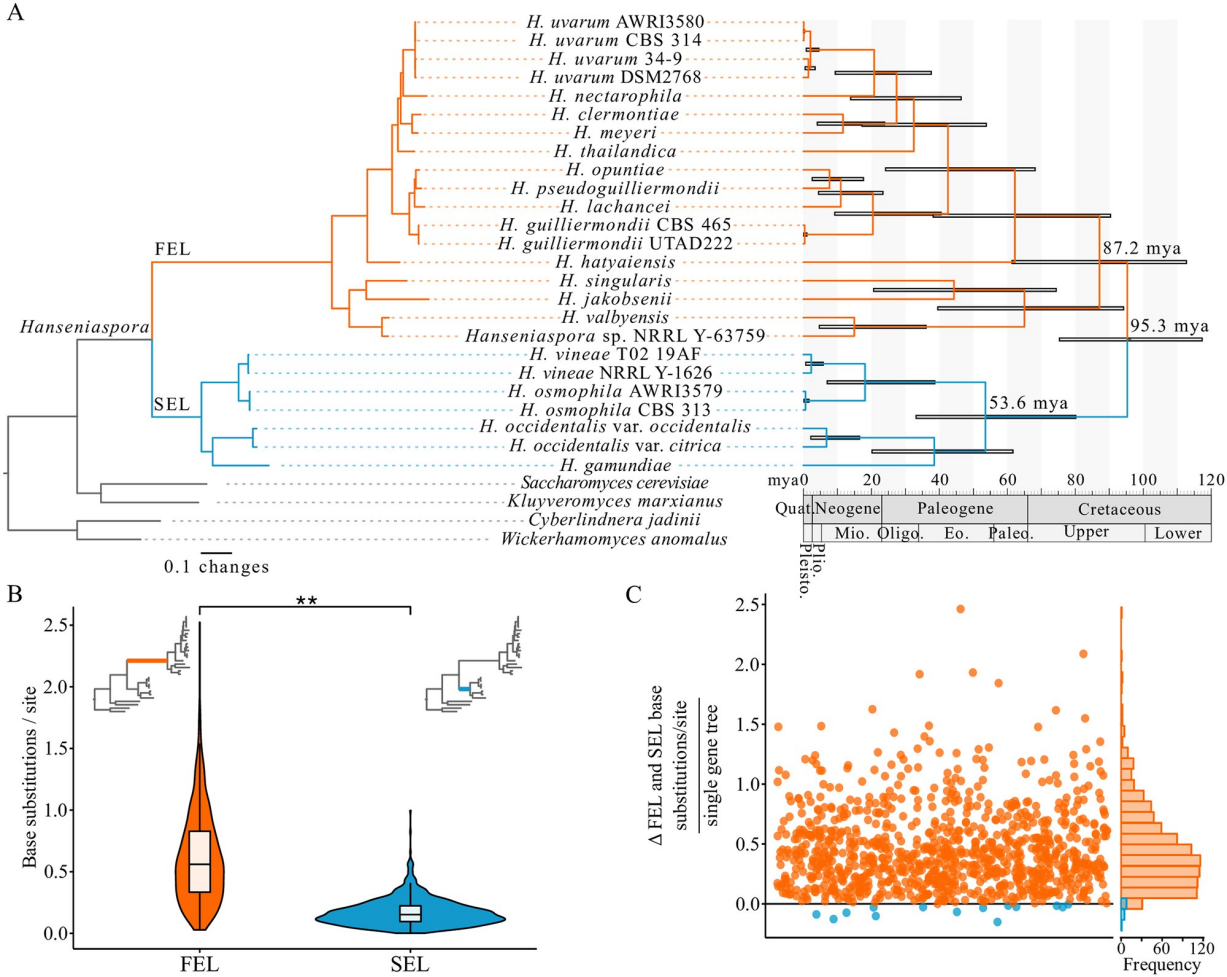
The evolutionary history, rate, and timeline of *Hanseniaspora* diversification. (A) Phylogenomic and relaxed molecular clock analysis of 1,034 single-copy OGs from a near-complete set of *Hanseniaspora* species revealed two well-supported lineages termed the FEL and SEL, which began diversifying around 87.2 and 53.6 mya after diverging 95.3 mya. (B) Among single-gene phylogenies in which the FEL and SEL were monophyletic (*n* = 946), the FEL stem branch was consistently and significantly longer (0.62 ± 0.38 base substitutions/site) than the SEL stem branch (0.17 ± 0.11 base substitutions/site) (*p* < 0.001; paired Wilcoxon rank–sum test). (C) Examination of the difference between FEL and SEL: stem branch lengths per single-gene tree revealed that 932 single-gene phylogenies had a longer FEL stem branch (depicted in orange with values greater than 0), while only 14 single-gene phylogenies had a longer SEL stem branch (depicted in blue with values less than 0). Across all single-gene phylogenies, the average difference in stem branch length between the two lineages was 0.45. figshare: https://doi.org/10.6084/m9.figshare.7670756.v2. AWRI, Australian Wine Research Institute; CBS, Centraalbureau voor Schimmelcultures; DSM2768, Dutch State Mines 2768; Eo., Eocene; FEL, faster-evolving lineage; Mio., Miocene; mya, million years ago; NRRL, Northern Regional Research Laboratory; OG, orthologous gene; Oligo., Oligocene; Paleo., Paleocene; Pleisto., Pleistocene; Plio., Pliocene; Quat., Quaternary; SEL, slower-evolving lineage; UTAD222, University of Trás-os-Montes and Alto Douro 222.

Concatenation and coalescence analyses of a data matrix of 1,034 single-copy orthologous genes (OGs) (522,832 sites; 100% taxon-occupancy) yielded a robust phylogeny of the genus *Hanseniaspora* (Fig 1A, S1 and S2 Figs). Consistent with previous analyses [35,36,42], our phylogeny identified two major lineages, each of which had a long stem branch; we hereafter refer to the lineage with the longer stem branch as the FEL and to the other as the SEL. Relaxed molecular clock analysis suggests that the FEL and SEL split 95.34 (95% credible interval [CI]: 117.38–75.36) mya, with the origin of their crown groups estimated at 87.16 (95% CI: 112.75–61.38) and 53.59 (95% CI: 80.21–33.17) mya, respectively (Fig 1A, S3 Fig and S2 File).

The FEL stem branch is much longer than the SEL stem branch in the *Hanseniaspora* phylogeny (Fig 1) (see also phylogenies in [35,36]). To determine whether this difference in branch length was a property of some or all single-gene phylogenies, we compared the difference in length of the FEL and SEL stem branches among all single-gene trees in which each lineage was inferred to be monophyletic (*n* = 946). We found that the FEL stem branch was nearly four times longer (0.62 ± 0.38 substitutions/site) than the SEL stem branch (0.17 ± 0.11 substitutions/site) (Fig 1B; *p* < 0.001; paired Wilcoxon rank–sum test). Furthermore, of the 946 gene trees examined, 932 had a much longer FEL stem branch (0.46 ± 0.33 Δ substitutions/site), whereas only 14 had a slightly longer SEL stem branch (0.06 ± 0.05 Δ substitutions/site).

### The genomes of FEL species have lost substantial numbers of genes

Examination of Guanine–Cytosine (GC) content, genome size, and gene number revealed that the some of the lowest GC content values, as well as the smallest genomes and lowest gene numbers, across the subphylum Saccharomycotina are primarily observed in FEL yeasts (S4 Fig). Specifically, the average GC contents for FEL yeasts (33.10 ± 3.53%), SEL yeasts (37.28 ± 2.05%), and all other Saccharomycotina yeasts (40.77 ± 5.58%) are significantly different from one another (χ^2^(2) = 30.00, *p* < 0.001; Kruskal–Wallis rank–sum test). Pairwise comparisons of GC contents between the FEL, SEL, and all other Saccharomycotina were not significant, except in the comparison between the FEL and other Saccharomycotina yeasts (*p* < 0.001; Dunn’s test for multiple comparisons with Benjamini–Hochberg multitest correction).

For genome size and gene number, FEL yeast genomes have average sizes of 9.71 ± 1.32 Mb and contain 4,707.89 ± 633.56 genes, respectively, while SEL yeast genomes have average sizes of 10.99 ± 1.66 Mb and contain 4,932.43 ± 289.71 genes. In contrast, all other Saccharomycotina have average genome sizes and gene numbers of 13.01 ± 3.20 Mb and 5,726.10 ± 1,042.60, respectively. Statistically significant differences were observed between the FEL, SEL, and all other Saccharomycotina (genome size: χ^2^(2) = 33.47, *p* < 0.001 and gene number: χ^2^(2) = 31.52, *p* < 0.001; Kruskal–Wallis rank–sum test for both). Pairwise comparisons of genome size and gene number between the FEL, SEL, and all other Saccharomycotina revealed that the only significant difference for genome size was between the FEL and other Saccharomycotina yeasts (*p* < 0.001; Dunn’s test for multiple comparisons with Benjamini–Hochberg multitest correction), while both the FEL and SEL had smaller gene sets compared to other Saccharomycotina yeasts (*p* < 0.001 and *p* = 0.008, respectively; Dunn’s test for multiple comparisons with Benjamini–Hochberg multitest correction). The lower numbers of genes in the FEL (especially) and SEL lineages were also supported by gene-content completeness analyses using orthologous sets of genes constructed from sets of genomes representing multiple taxonomic levels across eukaryotes (S5 Fig) from the orthoDB database [43].

**Fig 2:**
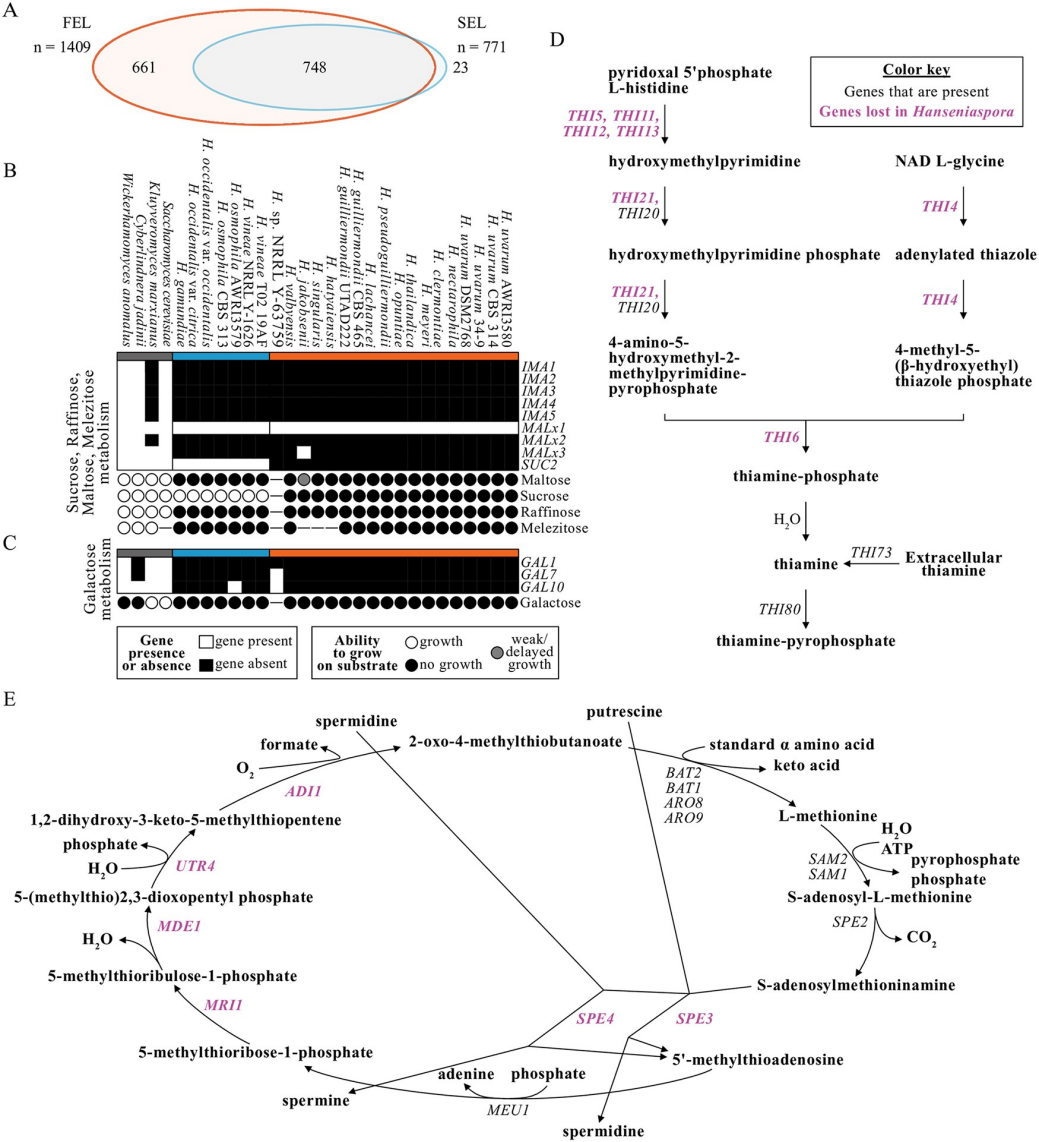
Gene presence and absence analyses reflect phenotype and reveal disrupted pathways. (A) Examination of gene presence and absence (see Methods) revealed numerous genes that were lost across *Hanseniaspora*. Specifically, 1,409 were lost in the FEL, and 771 genes were lost in the SEL. A Euler diagram represents the overlap of these gene sets. Both lineages have lost 748 genes, the FEL has lost an additional 661, and the SEL has lost an additional 23. (B) The *IMA* gene family (*IMA1*–*5*) encoding α-glucosidases, *MAL* (*MALx1*–*3*) loci, and *SUC2* are associated with growth on maltose, sucrose, raffinose, and melezitose. The *IMA* and *MAL* loci are largely absent among *Hanseniaspora* with the exception of homologs *MALx1*, which encode diverse transporters of the major facilitator superfamily whose functions are difficult to predict from sequence; as expected, *Hanseniaspora* spp. cannot grow on maltose, raffinose, and melezitose, with the sole exception of *H*. *jakobsenii*, which has delayed/weak growth on maltose and is the only *Hanseniaspora* species with *MALx3*, which encodes a homolog of the *MAL*-activator protein. (C) The genes involved with galactose degradation are largely absent among *Hanseniaspora* species, which correlates with their inability to grow on galactose. Genes that are present are depicted in white, and genes that are absent are depicted in black. The ability to grow, the ability to weakly grow/exhibit delayed growth on a given substrate, or the inability to grow is specified using white, gray, and black circles, respectively; dashes indicate no data. (D) Most genes involved in the thiamine biosynthesis pathway are absent among all *Hanseniaspora*. (E) Many genes involved in the methionine salvage pathway are absent among all *Hanseniaspora*. Absent genes are depicted in purple. figshare: https://doi.org/10.6084/m9.figshare.7670756.v2. *ADI*, Acireductone Dioxygenase; *ARO*, AROmatic amino-acid requiring; AWRI, Australian Wine Research Institute; *BAT*, Branched-chain Amino-acid Transaminase; CBS, Centraalbureau voor Schimmelcultures; DSM2768, Dutch State Mines 2768; FEL, faster-evolving lineage; *GAL*, GALactose metabolism; *IMA*, IsoMAltase; *MAL*, MALtose fermentation; *MDE*, Methylthioribulose-1-phosphate DEhydratase; *MEU*, Multicopy Enhancer of Upstream activation site; *MRI*, MethylthioRibose-1-phosphate Isomerase; NRRL, Northern Regional Research Laboratory; *SAM*, S-AdenosylMethionine requiring; SEL, slower-evolving lineage; *SUC2*, SUCrose; *THI*, THIamine regulon; UTAD222, University of Trás-os-Montes and Alto Douro 222; *UTR*, Unidentified Transcript.

To further examine which genes have been lost in the genomes of FEL and SEL species relative to other representative Saccharomycotina genomes, we conducted Hidden Markov Model (HMM)-based sequence similarity searches using annotated *S*. *cerevisiae* genes as queries in HMM construction (see Methods) (S6 Fig). Because we were most interested in broad patterns of gene losses in the FEL and SEL, we focused our analyses on genes lost in at least two-thirds of each lineage (i.e., ≥ 11 FEL taxa or ≥ 5 SEL taxa). Using this criterion, we found that 1,409 and 771 genes have been lost in the FEL and SEL, respectively (Fig 2A). Among the genes lost in each lineage, 748 genes were lost across both lineages, 661 genes were uniquely lost in the FEL, and 23 genes were uniquely lost in the SEL (S3 File).

To identify the likely functions of genes lost from each lineage, we conducted gene ontology (GO) enrichment analyses. Examination of significantly over-represented GO terms for the sets of genes that have been lost in *Hanseniaspora* genomes revealed numerous categories related to metabolism (e.g., maltose metabolic process, GO:0000023, *p* = 0.006; sucrose alpha-glucosidase activity, GO:0004575, *p* = 0.003) and genome-maintenance processes (e.g., meiotic cell cycle, GO:0051321, *p* < 0.001) (S4 File). Additional terms, such as cell cycle, GO:0007049 (*p* < 0.001), chromosome segregation, GO:0007059 (*p* < 0.001), chromosome organization, GO:0051276 (*p* = 0.009), and DNA-directed DNA polymerase activity, GO:0003887 (*p* < 0.001), were significantly over-represented among genes absent only in the FEL. Next, we examined in more detail the identities and likely functional consequences of extensive gene losses across *Hanseniaspora* associated with metabolism, the cell cycle, and DNA repair.

#### Metabolism-associated gene losses

Examination of the genes causing over-representation of metabolism-associated GO terms revealed gene losses in the IsoMAltase (*IMA*) gene family and the MALtose fermentation (*MAL*) loci, both of which are associated with growth primarily on maltose but can also facilitate growth on sucrose, raffinose, and melezitose [44,45]. All *IMA* genes have been lost in *Hanseniaspora*, whereas MALtose fermentation locus 3 (*MALx3*), which encodes the *MAL*-activator protein [46], has been lost in all but one species (*H*. *jakobsenii*; Fig 2B). Consistent with these losses, *Hanseniaspora* species cannot grow on the carbon substrates associated with these genes (i.e., maltose, raffinose, and melezitose) with the exception of *H*. *jakobsenii*, which has weak/delayed growth on maltose (Fig 2B and S5 File). The growth of *H*. *jakobsenii* on maltose may be due to a cryptic α-glucosidase gene or represent a false positive because *MALx2* encodes the required enzyme for growth on maltose and is absent in *H*. *jakobsenii*. Because these genes are also associated with growth on sucrose in some species [44], we also examined their ability to grow on this substrate. In addition to the *MAL* loci conferring growth on sucrose, the invertase SUCrose 2 (Suc2) can also break down sucrose into glucose and fructose [47]. We found that FEL yeasts have lost *SUC2* and are unable to grow on sucrose, while SEL yeasts have *SUC2* and are able to grow on this substrate (Fig 2B and S5 File). Altogether, patterns of gene loss are consistent with known metabolic traits.

Examination of gene sets associated with growth on other carbon substrates revealed that *Hanseniaspora* species also cannot grow on galactose, consistent with the loss of one or more of the three genes involved in galactose assimilation (GALactose metabolism 1 [*GAL1*], *GAL7*, and *GAL10*) from their genomes (Fig 2C and S5 File). Additionally, all *Hanseniaspora* genomes appear to have lost two key genes, Phosphoenolpyruvate CarboxyKinase 1 (*PCK1*) and Fructose-1,6-BisPhosphatase 1 (*FBP1*), encoding enzymes in the gluconeogenesis pathway (S7A Fig); in contrast, all *Hanseniaspora* have an intact glycolysis pathway (S7B Fig).

Altogether these metabolism-associated gene losses may reflect *Hanseniaspora* ecology. More specifically, among wine strains of *S*. *cerevisiae*, genes associated with maltose and thiamine metabolism are frequently absent in their genomes [48,49] and are thought to reflect their ecology in the grape must environment [50]. Interestingly, similar gene losses are observed among *Hanseniaspora* species but are often more pronounced; e.g., *Hanseniaspora* species lack most of the thiamine biosynthesis pathway, while wine strains of *S*. *cerevisiae* typically lack a single member of the THIamine regulon (*THI*) gene family.

Manual examination of other metabolic pathways revealed that *Hanseniaspora* genomes are also lacking some of their key genes. E.g., we found that thiamine biosynthetic process, GO:0009228 (*p* = 0.003), was an over-represented GO term among genes absent in both the FEL and SEL because of the absence of *THI* and SNooze proximal Open reading frame (*SNO*) family genes. Further examination of genes present in the thiamine biosynthesis pathway revealed extensive gene loss (Fig 2D), which is consistent with their inability to grow on vitamin-free media [45] (S5 File). Notably, *Hanseniaspora* are still predicted to be able to import extracellular thiamine via Thi73 and convert it to its active cofactor via Thi80, which may explain why they can rapidly consume thiamine [40]. Similarly, examination of amino-acid biosynthesis pathways revealed the methionine salvage pathway was also largely disrupted by gene losses across all *Hanseniaspora* (Fig 2E). Lastly, we found that Glutamate DeHydrogenase 1 (*GDH1*) and Glutamate DeHydrogenase 3 (*GDH3*) from the glutamate biosynthesis pathway from ammonium are absent in FEL yeasts (S3 File). However, *Hanseniaspora* have GLuTamate synthase 1 (*GLT1*), which enables glutamate biosynthesis from glutamine.

#### Cell-cycle– and genome-integrity–associated gene losses

**Fig 3:**
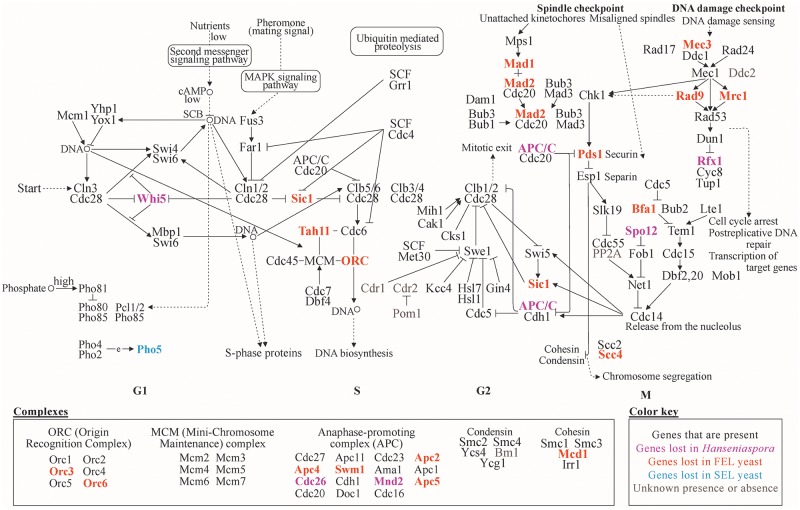
Gene presence and absence in the budding yeast cell cycle. Examination of cell-cycle genes revealed numerous genes that are absent in *Hanseniaspora* genomes. The genes not present in *Hanseniaspora* participate in diverse functions and include key regulators such as *WHI5*, components of spindle checkpoint processes and segregation such as *MAD1* and *MAD2*, and components of DNA-damage–checkpoint processes such as *MEC3*, *RAD9*, and *RFX1*. Genes absent in both lineages, the FEL, or the SEL are colored purple, orange, or blue, respectively. The “e” in the PHO cascade represents expression of Pho4:Pho2. Dotted lines with arrows indicate indirect links or unknown reactions. Lines with arrows indicate molecular interactions or relations. Circles indicate chemical compounds such as DNA. figshare: https://doi.org/10.6084/m9.figshare.7670756.v2. Ama, Activator of meiotic anaphase-promoting complex; APC (or APC/C), Anaphase-Promoting Complex; Bfa, Byr-four-alike; Bm1, Biomimetic moiety glutathionesulfonic acid; Bub, Budding uninhibited by benzimidazole; Cak1, Cyclin-dependent kinase-activating kinase; cAMP, cyclic AdenosineMonoPhosphate; Cdc, Cell division cycle; Cdh, CDC20 homolog; Cdr, *Candida* drug resistance; Chk, Checkpoint kinase; Cks, Cdc28 kinase subunit; Clb, Cyclin B; Cln, Cyclin; Cyc, Cytochrome C; Dam, Duo1 and Mps1 interacting; Dbf, Dumbbell former; Ddc, DNA Damage Checkpoint; Doc, Destruction of Cyclin B; Dun, DNA-damage UNinducible; Esp1, Extra spindle pole bodies 1; Far1, Factor ARrest; FEL, faster-evolving lineage; Fob, Fork Blocking less; Fus3, cell fusion 3; Gin4, Growth inhibitory 4; Grr, Glucose repression-resistant; Hsl, Histone synthetic lethal; Irr, Irregular cell behavior; Kcc, K^+^-Cl^−^ cotransporters; Lte, Low temperature essential; *MAD*, Mitotic Arrest-Deficient; MAPK, Mitogen-Activated Protein Kinase; Mbp, Mlul-box–binding protein; Mcd, Mitotic chromosome determinant; MCM, Mini-Chromosome Maintenance; *MEC3*, Mitosis Entry Checkpoint 3; Met30, Methionine requiring 30; Mih1, Mitotic inducer homolog; Mnd, Meiotic nuclear divisions; Mob, Mps one binder; Mps, Monopolar spindle; Mrc, Mediator of the Replication Checkpoint; Net, Nucleolar silencing establishing factor and telophase regulator; ORC, Origin Recognition Complex; Pds, Precocious Dissociation of Sisters; PHO, PHOsphate; Pom, Polarity misplaced; PP2A, Protein Phosphatase 2A; *RAD9*, RADiation sensitive; *RFX1*,; SCB, Swi4,6-dependent cell ycle box; Scc, Sister Chromatid Cohesion; SCF, S-phase kinase-associated protein, Cullin, F-box containing complex; SEL, slower-evolving lineage; Sic, Sucrose NonFermenting; Slk, Synthetic lethal karyogamy; Smc, Stability of minichromosomes; Spo, Sporulation; Swe, *Saccharomyces* Wee1; Swi, Switching deficient; Swm, Spore Wall Maturation; Tah11, Topo-A Hypersensitive; Tem, Termination of M phase; Tup, deoxythymidine monophosphate-uptake; *WHI5*, WHIskey 5; Ycg, Yeast cap G; Ycs, Yeast condensing subunit; Yhp1, Yeast Homeo-Protein 1; Yox1, Yeast homeobox 1.

Many genes involved in the cell cycle and genome integrity, including cell-cycle checkpoint genes, have been lost across *Hanseniaspora* (Fig 3). E.g., *WHI5* and Daughter-Specific Expression 2 (*DSE2*), which are responsible for repressing the start (i.e., an event that determines cells have reached a critical size before beginning division) [51] and help facilitate daughter–mother cell separation through cell wall degradation [52], have been lost in both lineages. Additionally, the FEL has lost the entirety of the Dam1 complex (or DASH complex) (i.e., Associated with Spindles and Kinetochores 1 [*ASK1*], Death Upon Overproduction 1 [*DUO1*] And Duo1 and MonoPolar Spindle 1 [*MPS1*] [*DAM1*] interacting [DAD] 1, *DAD2*, *DAD3*, *DAD4*, *DUO1*, *DAM1*, Helper of ASK1 3 [*HSK3*], Spindle Pole Component [SPC] 19, and *SPC34*), which forms part of the kinetochore and functions in spindle attachment and stability as well as chromosome segregation, and the Mis TWelve-like 1 (Mtw1) protein Including Necessary for Nuclear Function 1 (Nnf1) protein–Nnf1 Synthetic Lethal 1 (Nsl1) protein–Dosage Suppressor of NNF1 (Dsn1) protein (MIND) complex (i.e., *MTW1*, *NNF1*, *NSL1*, and *DSN1*), which is required for kinetochore biorientation and accurate chromosome segregation (S3 and S4 Files). Similarly, FEL species have lost *MAD1* and *MAD2*, which are associated with spindle checkpoint processes and have abolished checkpoint activity when their encoded proteins are unable to dimerize [14]. Lastly, components of the APC, a major multi-subunit regulator of the cell cycle, are lost in both lineages (i.e., *CDC26* and Meiotic Nuclear Divisions 2 [*MND2*]) or just the FEL (i.e., *APC2*, *APC4*, *APC5*, and Spore Wall Maturation 1 [*SWM1*]).

Another group of genes that have been lost in *Hanseniaspora* are genes associated with the DNA damage checkpoint and DNA damage sensing. E.g., both lineages have lost Regulatory Factor X1 (*RFX1*), which controls a late point in the DNA-damage–checkpoint pathway [53], whereas the FEL has lost *MEC3* and *RAD9*, which encode checkpoint proteins required for arrest in the G2 phase after DNA damage has occurred [17]. Since losses in DNA damage checkpoints and dysregulation of spindle checkpoint processes are associated with genomic instability, we next evaluated the ploidy of *Hanseniaspora* genomes [54]. Using base frequency plots, we found that the ploidy of genomes of FEL species ranges between 1 and 3, with evidence suggesting that certain species—such as *H*. *singularis*, *H*. *pseudoguilliermondii*, and *H*. *jakobsenii*—are potentially aneuploid (S8 Fig). In contrast, the genomes of SEL species have ploidies of 1–2 with evidence of potential aneuploidy observed only in *H*. *occidentalis* var. *citrica*. Greater variance in ploidy and aneuploidy in the FEL compared to the SEL may be due to the FEL’s loss of a greater number of components of the APC, whose dysregulation is thought to increase instances of aneuploidy [55].

Lastly, we examined losses among genes related to meiosis. Although little is known about meiosis and sexual reproduction in *Hanseniaspora*, recent attempts to induce sporulation and sexual reproduction in different *Hanseniaspora* species have been unsuccessful [37,41,56,57]. In contrast, other species (i.e., *H*. *thailandica*, *H*. *singularis*, and *H*. *gamundiae*) are able to sporulate [42,58]. These inconsistences may be due to the infrequency of sporulation or reduced total number of spores produced, which may be linked to the losses of genes associated with coordinating meiosis such as the major regulator Inducer of MEiosis 1 (*IME1*) [59] and genes associated with spore formation such as Sporulation-specific protein 1 (*SSP1*) [60] and Glycogen 7-Interacting Protein 1 (*GIP1*) [61] (S9 Fig).

#### Pronounced losses of DNA repair genes in the FEL

**Fig 4:**
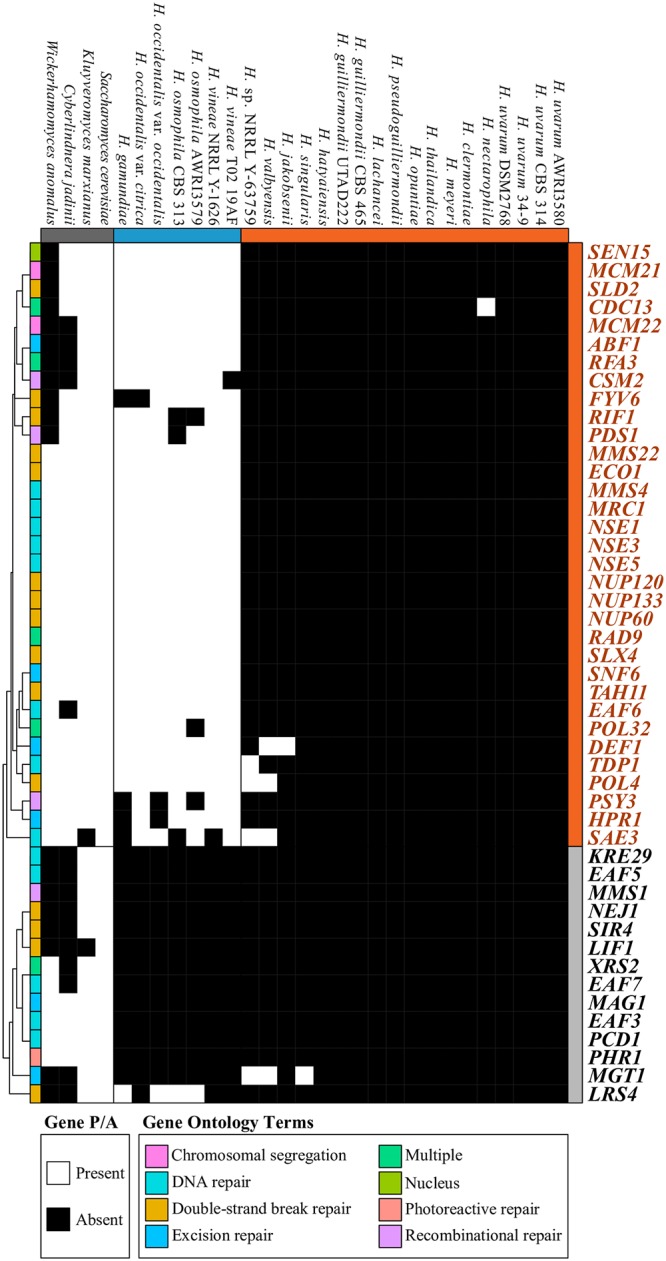
A panoply of genome-maintenance and DNA repair genes are absent among *Hanseniaspora*, especially in the FEL. Genes annotated as DNA repair genes according to GO (GO:0006281) and child terms were examined for presence and absence in at least two-thirds of each lineage, respectively (268 total genes). 47 genes are absent among the FEL species, and 14 genes are absent among the SEL. Presence and absence of genes was clustered using hierarchical clustering (cladogram on the left) where each gene’s ontology is provided as well. Genes with multiple gene annotations are denoted as such using the “multiple” term. figshare: https://doi.org/10.6084/m9.figshare.7670756.v2. *ABF1*, Autonomously replicating sequence-Binding Factor 1; AWRI, Australian Wine Research Institute; CBS, Centraalbureau voor Schimmelcultures; *CDC13*, Cell Division Cycle 13; *CSM2*, Chromosome Segregation in Meiosis 2; *DEF1*, RNA polymerase II Degradation Factor 1; DSM2768, Dutch State Mines 2768; *EAF6*, Essential something about silencing 2-related acetyltransferase 1-Associated Factor 6; *ECO1*, Establishment of Cohesion 1; FEL, faster-evolving lineage; *FYV6*, Function required for Yeast Viability 6; GO, gene ontology; *HPR1*, HyPerRecombination 1; *KRE29*, Killer toxin Resistant 29; *LIF1*, Ligase Interacting Factor 1; *LRS4*, Loss of RDNA Silencing 4; *MAG1*, 3-MethylAdenine DNA Glycosylase 1; *MCM21*, Mini-Chromosome Maintenance 21; *MGT1*, O-6-MethylGuanine-DNA methylTransferase 1; *MMS22*, Methyl MethaneSulfonate sensitivity 22; *MRC1*, Mediator of the Replication Checkpoint 1; *NEJ1*, Nonhomologous End-Joining defective 1; NRRL, Northern Regional Research Laboratory; *NSE1*, NonStructural maintenance of chromosomes Element 1; *NUP120*, NUclear Pore 120; *PCD1*, Peroxisomal Coenzyme A Diphosphatase 1; *PDS1*, Precocious Dissociation of Sisters 1; *PHR1*, PHotoreactivation Repair deficient 1; *POL32*, POLymerase 32; *PSY3*, Platinum SensitivitY 3; P/A, presence or absence; *RAD9*, RADiation sensitive 9; *RFA3*, Replication Factor A 3; *RIF1*, Repressor/activator site binding protein-Interacting Factor 1; *SAE3*, Sporulation in the Absence of sporulation Eleven; SEL, slower-evolving lineage; *SEN15*, Splicing ENdonuclease 15; *SIR4*, Silent Information Regulator 4; *SLD2*, Synthetically Lethal with DNA polymerase B (II)-1 2; *SLX4*, Synthetical Lethal of unknown (X) function 4; *SNF6*, Sucrose NonFermenting 6; *TAH11*, Topo-A Hypersensitive 11; *TDP1*, Tyrosyl-DNA Phosphodiesterase 1;UTAD222, University of Trás-os-Montes and Alto Douro 222; *XRS2*, X-Ray Sensitive 2.

Examination of other GO-enriched terms revealed numerous genes associated with diverse DNA repair processes that have been lost among *Hanseniaspora* species, and especially the FEL (Fig 4). We noted 14 lost DNA repair genes across all *Hanseniaspora*, including the DNA glycosylase gene *MAG1* [62], the photolyase gene *PHR1* that exclusively repairs pyrimidine dimers [23], and the diphosphatase gene *PCD1*, a key contributor to the purging of mutagenic nucleotides, such as 8-oxo-dGTP, from the cell [24]. An additional 33 genes were lost specifically in the FEL such as Tyrosyl-DNA Phosphodiesterase 1 (*TDP1*), which repairs damage caused by topoisomerase activity [63]; the DNA polymerase gene *POL32*, which participates in base-excision and nucleotide-excision repair and whose null mutants have increased genomic deletions [20]; and the *CDC13* gene, which encodes a telomere-capping protein [64].

### FEL gene losses are associated with accelerated sequence evolution

#### Loss of DNA repair genes is associated with a burst of sequence evolution

**Fig 5:**
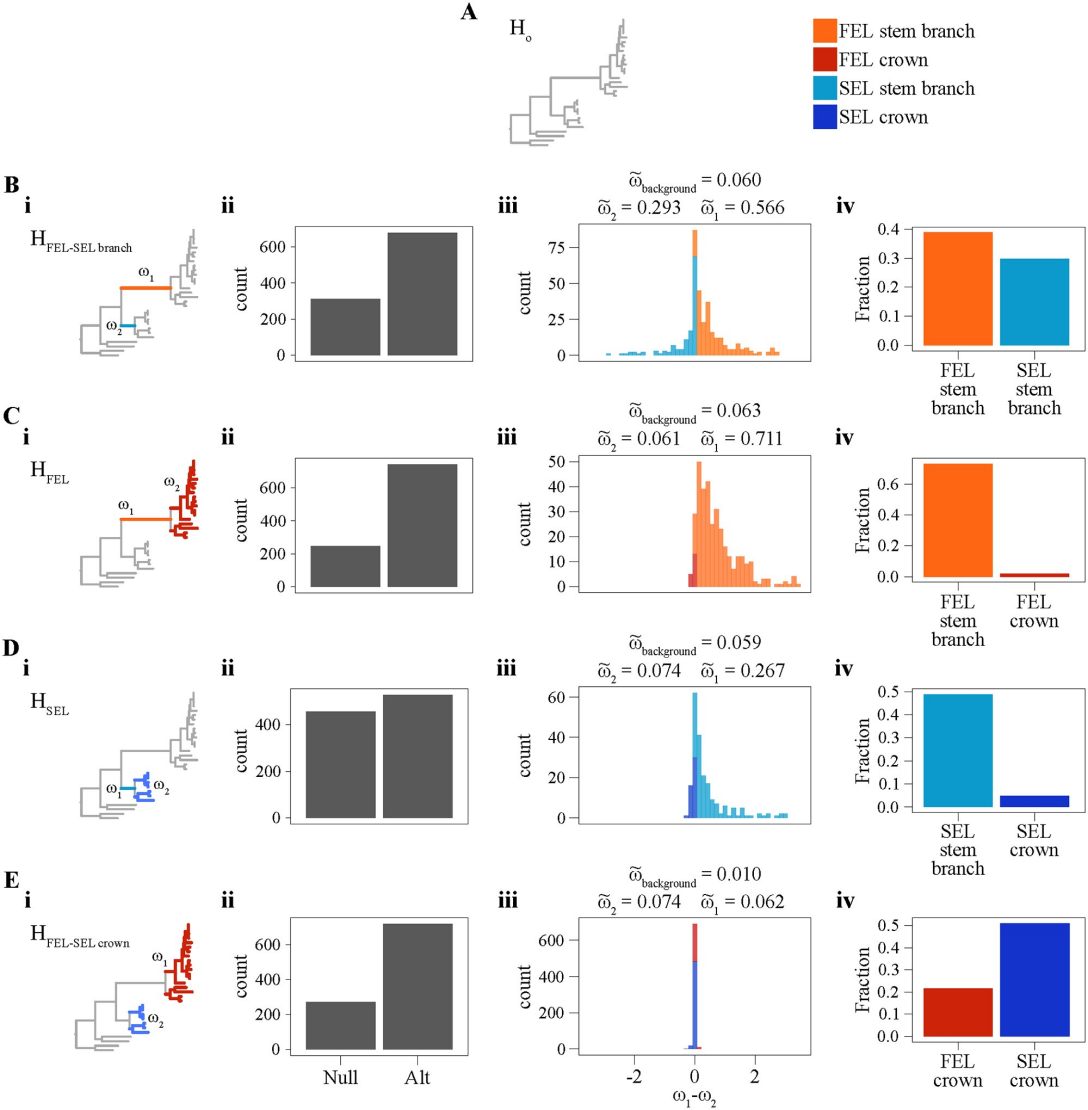
dN/dS (ω) analyses support a historical burst of accelerated evolution in the FEL. (A) The null hypothesis (H_O_) that all branches in the phylogeny have the same ω value. Alternative hypotheses (B–E) evaluate ω along three sets of branches. (Bi) The alternative hypothesis (H_FEL–SEL branch_) examined ω values along the FEL and SEL stem branches. (Bii) 311 (31.45%) genes supported H_O_, and 678 (68.55%) genes supported H_FEL–SEL branch_. (Biii) Among the genes that supported H_FEL–SEL branch_, we examined the distribution of the difference between ω_1_ and ω_2_ as specified in part Bi. Here, a range of ω_1_–ω_2_ of −3.5 to 3.5 is shown in the histogram. Additionally, we report the median ω_1_ and ω_2_ values, which are 0.57 and 0.29, respectively. (Biv) 384 (38.83%) genes significantly rejected H_O_ and were faster in the FEL than the SEL, while 237 (23.96%) significantly rejected H_O_ and were faster in the SEL than the FEL. (Ci) The alternative hypothesis (H_FEL_) examined ω values along the FEL stem branch (ω_1_) and crown branches (ω_2_). (Cii) 246 (24.87%) genes supported H_O_, and 743 (75.13%) genes supported H_FEL_. (Ciii) Among the genes that supported H_FEL_, we examined the distribution of the difference between ω_1_ and ω_2_ as specified in part Ci. The median ω_1_ and ω_2_ values were 0.71 and 0.06, respectively. (Civ) 725 (73.31%) genes significantly rejected H_O_ and had higher ω_1_ values than ω_2_ values, while 18 (1.82%) genes significantly rejected H_O_ and had higher ω_2_ than ω_1_ values. (Di) The alternative hypothesis (H_SEL_) examined ω values along the SEL stem branch (ω_1_) and crown branches (ω_2_). (Dii) 455 (46.29%) genes supported H_O_, and 528 (53.71%) genes supported H_SEL_. (Diii) Among the genes that supported H_SEL_, we examined the distribution of the difference between ω_1_ and ω_2_ as specified in part Di. The median ω_1_ and ω_2_ values were 0.27 and 0.07, respectively. (Div) 481 (48.93%) genes significantly rejected H_O_ and had higher ω_1_ than ω_2_ values, while 47 (4.78%) genes significantly rejected H_O_ and had higher ω_2_ than ω_1_ values. (Ei) The alternative hypothesis (H_FEL–SEL crown_) examined ω values in the FEL crown branches (ω_1_) and SEL crown branches (ω_2_). (Eii) 272 (27.50%) genes supported H_O_, and 717 (72.50%) genes supported H_FEL–SEL crown_. (Eiii) Among the genes that supported H_FEL–SEL crown_, we examined the distribution of the difference between ω_1_ and ω_2_ as specified in part Di. The median ω_1_ and ω_2_ values were 0.06 and 0.07, respectively. (Eiv) 481 (21.54%) genes significantly rejected H_O_ and had higher ω_1_ than ω_2_ values, while 504 (50.96%) genes had higher ω_2_ than ω_1_ values. figshare: https://doi.org/10.6084/m9.figshare.7670756.v2. dN, rate of nonsynonymous substitutions; dS, rate of synonymous subsitutions; FEL, faster-evolving lineage; SEL, slower-evolving lineage.

To examine the mutational signatures of losing numerous DNA repair genes on *Hanseniaspora* substitution rates, we tested several different hypotheses that postulated changes in the ratio of the rate of nonsynonymous (dN) to the rate of synonymous substitutions (dS) (dN/dS or ω) along the phylogeny (Table 1 and Fig 5). For each hypothesis tested, the null was that the ω value remained constant across all branches of the phylogeny. Examination of the hypothesis that the ω values of both the FEL and SEL stem branches were distinct from the background ω value (H_FEL–SEL branch_; Fig 5B), revealed that 678 genes (68.55% of examined genes) significantly rejected the null hypothesis (Table 1; α = 0.01; likelihood ratio test [LRT]; median FEL stem branch ω = 0.57, median SEL stem branch ω = 0.29, and median background ω = 0.060). Examination of the hypothesis that the ω value of the FEL stem branch and the ω value of the FEL crown branches were distinct from the background ω value (H_FEL_; Fig 5C) revealed 743 individual genes (75.13% of examined genes) that significantly rejected the null hypothesis (Table 1; α = 0.01; LRT; median FEL stem branch ω = 0.71, median FEL crown branches ω = 0.06, median background ω = 0.063). Testing the same hypothesis for the SEL (H_SEL_; Fig 5D) revealed 528 individual genes (53.7% of examined genes) that significantly rejected the null hypothesis (Table 1; α = 0.01; LRT; median SEL stem branch ω = 0.267, median SEL crown branches ω = 0.074, median background ω = 0.059). Finally, testing of the hypothesis that the FEL and SEL crown branches have ω values distinct from each other and the background (H_FEL–SEL crown_; Fig 5E) revealed 717 genes (72.5% of examined genes) that significantly rejected the null hypothesis (Table 1; α = 0.01; LRT; median FEL crown branches ω = 0.062, median SEL crown branches ω = 0.074, median background ω = 0.010). These results suggest a dramatic, genome-wide increase in evolutionary rate in the FEL stem branch (Fig 5B and 5C), which coincided with the loss of a large number of genes involved in DNA repair.

**Table 1:** Rate of sequence evolution hypotheses and results.

Hypotheses for Interlineage Comparisons	Parameters	Fraction of Genes Significantly Different from H_O_	Median ω Values		
			ω_background_	ω_1_	ω_2_
**H_O_: Uniform rate for all branches** Fig 5A	Single ω value	N/A	N/A	N/A	N/A
**H_FEL–SEL branch_: Unique rates for FEL and SEL stem** Fig 5B	ω_background_ ≠ ω_1_ ≠ ω_2_ ω_1_ = FEL stem branch ω_2_ = SEL stem branch	678 genes (68.55% of examined genes)	0.060	0.566	0.293
**H_FEL_: Unique rates for FEL stem and FEL crown** Fig 5C	ω_background_ ≠ ω_1_ ≠ ω_2_ ω_1_ = FEL stem branch ω_2_ = FEL crown branches	743 genes (75.13% of examined genes)	0.063	0.711	0.061
**H_SEL_: Unique rates for SEL stem and SEL crown** Fig 5D	ω_background_ ≠ ω_1_ ≠ ω_2_ ω_1_ = SEL stem b≠anch ω_2_ = SEL crown branches	528 genes (53.7% of examined genes)	0.059	0.267	0.074
**H_FEL–SEL crown_: Unique rates for FEL crown and SEL crown** Fig 5E	ω_background_ ≠ ω_1_ ≠ ω_2_ ω_1_ = FEL crown branches ω_2_ = SEL crown branches	717 genes (72.5% of examined genes)	0.010	0.062	0.074

**Abbreviations:** FEL, faster-evolving lineage; SEL, slower-evolving lineage.

#### The FEL has a greater number of base substitutions and indels

**Fig 6:**
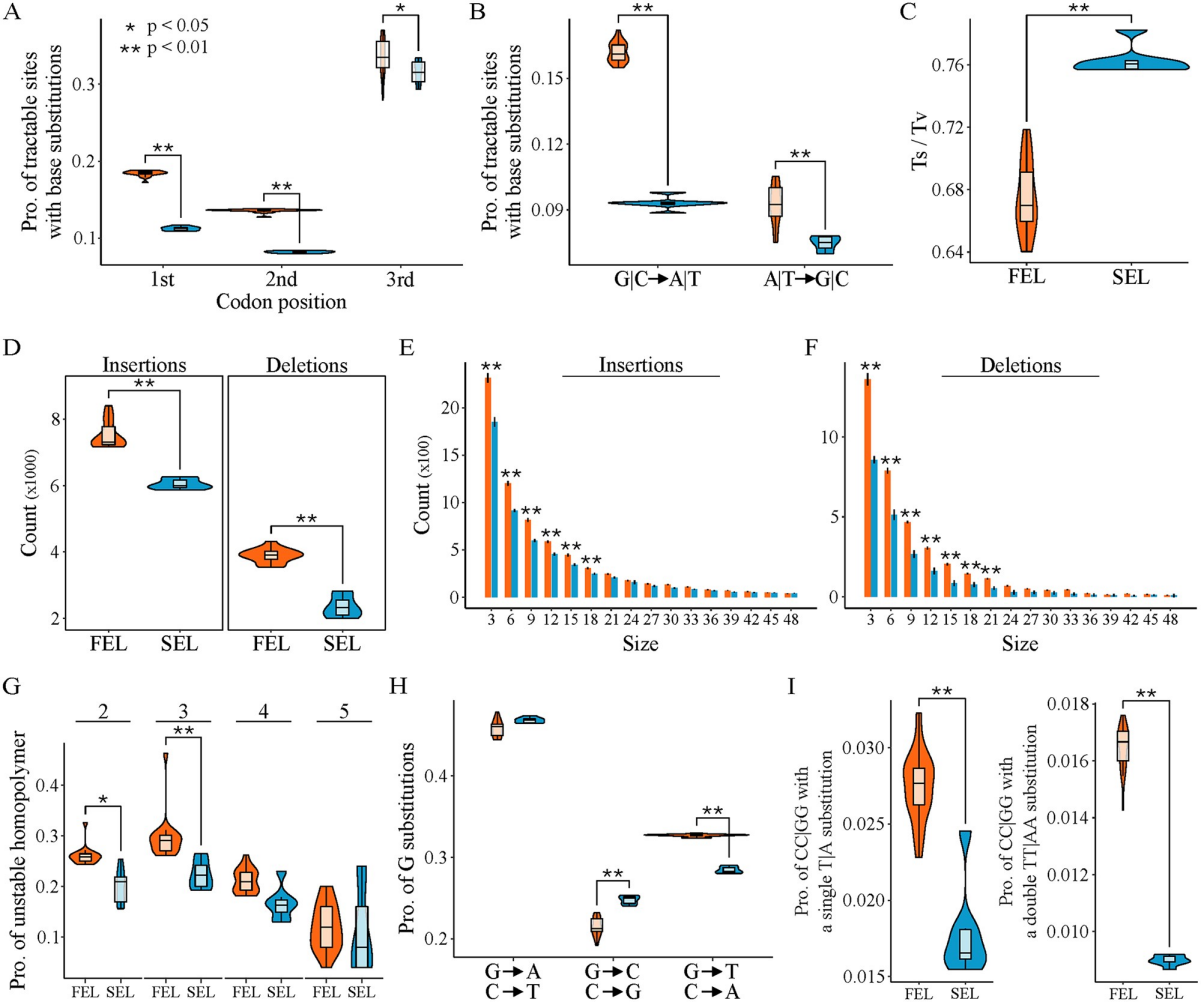
Analyses of base substitutions and indels reveal a higher mutational load in the FEL compared to the SEL. (A) Analyses of substitution patterns among codon-based alignments of 1,034 OGs revealed a higher number of base substitutions in the FEL compared to the SEL (F(1) = 196.88, *p* < 0.001; multifactor ANOVA) and an asymmetric distribution of base substitutions at codon sites (F(2) = 1,691.60, *p* < 0.001; multifactor ANOVA). A Tukey honest significance differences post hoc test revealed a higher proportion of substitutions in the FEL compared to the SEL at the first (*n* = 240,565; *p* < 0.001), second (*n* = 318,987; *p* < 0.001), and third (*n* = 58,151; *p* = 0.02) codon positions. (B) Analyses of the direction of base substitutions (i.e., G|C → A|T or A|T → G|C) revealed significant differences between the FEL and SEL (F(1) = 447.1, *p* < 0.001; multifactor ANOVA) as well as differences in the directionality of base substitutions (F(1) = 914.5, *p* < 0.001; multifactor ANOVA). A Tukey honest significance differences post hoc test revealed a significantly higher proportion of substitutions were G|C → A|T compared to A|T → G|C among sites that are G|C (*n* = 232,546) and A|T (*n* = 385,157) (*p* < 0.001), suggesting a general AT bias of base substitutions. Additionally, there was a significantly higher proportion of sites with base substitutions in the FEL compared to the SEL (*p* < 0.001). Specifically, a higher number of base substitutions was observed in the FEL compared to the SEL for both G|C → A|T (*p* < 0.001) and A|T → G|C mutations (*p* < 0.001), but the bias toward AT was greater in the FEL. (C) Examinations of transition/transversion ratios revealed a lower transition/transversion ratio in the FEL compared to the SEL (*p* < 0.001; Wilcoxon rank–sum test). (D) Comparisons of insertions and deletions revealed a significantly greater number of insertions (*p* < 0.001; Wilcoxon rank–sum test) and deletions (*p* < 0.001; Wilcoxon rank–sum test) in the FEL (${\bar{\text{X}}}_{\text{insertions}}=7,521.11\pm 405.34$; ${\bar{\text{X}}}_{\text{deletions}}=3,894.11\pm 208.16$) compared to the SEL (${\bar{\text{X}}}_{\text{insertions}}=6,049.571\pm 155.85$; ${\bar{\text{X}}}_{\text{deletions}}=2,346.71\pm 326.22$). (E and F) When adding the factor of size per insertion or deletion, significant differences were still observed between the lineages (F(1) = 2,102.87, *p* < 0.001; multifactor ANOVA). A Tukey honest significance differences post hoc test revealed that most differences were caused by significantly more small insertions and deletions in the FEL compared to the SEL. More specifically, there were significantly more insertions in the FEL compared to the SEL for sizes 3–18 (*p* < 0.001 for all comparisons between each lineage for each insertion size), and there were significantly more deletions in the FEL compared to the SEL for sizes 3–21 (*p* < 0.001 for all comparisons between each lineage for each deletion size). Black lines at the top of each bar show the 95% confidence interval for the number of insertions or deletions for a given size. (G) Evolutionarily conserved homopolymers of sequence length 2 (*n* = 17,391), 3 (*n* = 1,062), 4 (*n* = 104), and 5 (*n* = 5) were examined for substitutions and indels. Statistically significant differences of the proportion mutated bases (i.e., [base substitutions + deleted bases + inserted bases]/total homopolymer bases) were observed between the FEL and SEL (F(1) = 27.68, *p* < 0.001; multifactor ANOVA). Although the FEL had more mutations than the SEL for all homopolymers, a Tukey honest significance differences post hoc test revealed differences were statistically significant for homopolymers of two (*p* = 0.02) and three (*p* = 0.003). Analyses of homopolymers using additional factors of mutation type (i.e., base substitution, insertion, deletion) and homopolymer sequence type (i.e., A|T and C|G homopolymers) can be seen in S10 Fig. (H) G → T or C → A mutations are associated with the common and abundant oxidatively damaged base, 8-oxo-dG. When examining all substituted G positions for each species and their substitution direction, we found significant differences between different substitution directions (F(2) = 5,682, *p* < 0.001; multifactor ANOVA). More importantly, a Tukey honest significance differences post hoc test revealed an over-representation of G → T or C → A in the FEL compared to the SEL (*p* < 0.001). (I) Signatures of UV-damage–associated single and double substitutions (i.e., C → T at CC sites and CC → TT) double substitutions are greater in the FEL compared to the SEL (*p* < 0.001 for both tests; Wilcoxon rank–sum test). figshare: https://doi.org/10.6084/m9.figshare.7670756.v2. FEL, faster-evolving lineage; OG, orthologous gene; Pro., Proportion; SEL, slower-evolving lineage.

To better understand the mutational landscape in the FEL and SEL, we characterized patterns of base substitutions across the 1,034 OGs. Focusing on first (*n* = 240,565), second (*n* = 318,987), and third (*n* = 58,151) codon positions that had the same character state in all outgroup taxa, we first examined how many of these sites had experienced base substitutions in FEL and SEL species (Fig 6A). We found significant differences between the proportions of base substitutions in the FEL and SEL (F(1) = 196.88, *p* < 0.001; multifactor ANOVA) at each codon position (first: *p* < 0.001; second: *p* < 0.001; and third: *p* = 0.02; Tukey honest significance differences post hoc test).

We next investigated differences in the direction of substitutions. Specifically, we examined if substitutions were biased in the AT direction (i.e., G|C → A|T) or GC direction (i.e., A|T → G|C) as well as whether there are differences among substitutions in these directions between the FEL and the SEL. We observed significant differences among substitutions in the AT and GC directions between the FEL and the SEL (F(1) = 447.1, *p* < 0.001; multifactor ANOVA), as well as between overall AT and GC bias across both lineages among G|C (*n* = 232,546) and A|T (*n* = 385,157) sites (F(1) = 914.5, *p* < 0.001; multifactor ANOVA) (Fig 6B). There were significantly more base substitutions in the FEL compared to the SEL and a significant bias toward A|T across both lineages (*p* < 0.001 for both tests; Tukey honest significance differences post hoc test).

We next examined patterns of transition/transversion ratios and observed a lower transition/transversion ratio in the FEL (0.67 ± 0.02) compared to the SEL (0.76 ± 0.01) (Fig 6C; *p* < 0.001; Wilcoxon rank–sum test); this finding is in contrast to the transition/transversion ratios found in most known organisms, whose values are substantially above 1.00 [56–59]. Altogether, these analyses reveal more base substitutions in the FEL and SEL across all codon positions, a significant AT bias in base substitutions across all *Hanseniaspora*, and a low transition/transversion ratio across the FEL and SEL.

Examination of indels revealed that the total number of insertions or deletions was significantly greater in the FEL (mean_insertions_ = 7,521.11 ± 405.34; mean_deletions_ = 3,894.11 ± 208.16) compared to the SEL (mean_insertions_ = 6,049.571 ± 155.85; mean_deletions_ = 2,346.71 ± 326.22) (Fig 6D; *p* < 0.001 for both tests; Wilcoxon rank–sum test). The difference in number of indels between the FEL and SEL remained significant after taking into account indel size (F(1) = 2,102.87, *p* < 0.001; multifactor ANOVA). Further analyses revealed there are significantly more insertions in the FEL compared to the SEL for insertion sizes 3–18 bp (*p* < 0.001 for all comparisons between each lineage for each insertion size; Tukey honest significance differences post hoc test), while there were significantly more deletions in the FEL compared to the SEL for deletion sizes 3–21 bp (*p* < 0.001 for all comparisons between each lineage for each deletion size; Tukey honest significance differences post hoc test). These analyses suggest that there are significantly more indels in the FEL compared to the SEL and that this pattern is primarily driven by short indels.

### Greater sequence instability in the FEL and signatures of endogenous and exogenous DNA damage

#### The FEL has greater instability of homopolymers

Examination of the total proportion of mutated bases among homopolymers (i.e., stretches of the same base) in codon-based alignments of the 1,034 OGs (i.e., [substituted bases + deleted bases + inserted bases]/total homopolymer bases) revealed significant differences between the FEL and SEL (Fig 6G; F(1) = 27.68, *p* < 0.001; multifactor ANOVA). Although the FEL had a higher proportion of mutations among homopolymers across all sizes of two (*n* = 17,391), three (*n* = 1,062), four (*n* = 104), and five (*n* = 5), significant differences were observed for homopolymers of length two and three (*p* = 0.02 and *p* = 0.003, respectively; Tukey honest significance differences post hoc test). To gain more insight into the stability of different homopolymer runs (i.e., A|T or C|G) and the types of sequence changes that occur among homopolymers, we considered the additional factors of homopolymer sequence type (i.e., A|T or C|G) and mutation type (i.e., base substitution, insertion, or deletion) (S10 Fig). In addition to recapitulating differences between the types of mutations that occur at homopolymers (F(2) = 1,686.70, *p* < 0.001; multifactor ANOVA), we observed that base substitutions occurred more frequently than insertions and deletions (*p* < 0.001 for both tests; Tukey honest significance differences post hoc test). E.g., among A|T and C|G homopolymers of length 2 and C|G homopolymers of length 3, base substitutions were higher in the FEL compared to the SEL (*p* = 0.009, *p* < 0.001, and *p* < 0.001, respectively; Tukey honest significance differences post hoc test). Additionally, there were significantly more base substitutions in A|T homopolymers of length 5 in the FEL compared to the SEL (*p* < 0.001; Tukey honest significance differences post hoc test). Altogether, these analyses reveal greater instability of homopolymers in the FEL compared to the SEL because of more base substitutions.

#### The FEL has a stronger signature of endogenous DNA damage from 8-oxo-dG

Examination of mutational signatures associated with common endogenous and exogenous mutagens revealed greater signatures of mutational load in the FEL compared to the SEL, as well as in both FEL and SEL compared to the outgroup taxa. The oxidatively damaged guanine base, 8-oxo-dG, is a commonly observed endogenous form of DNA damage that causes the transversion mutation of G → T or C → A [26]. Examination of the direction of base substitutions among all sites with a G base in all outgroup taxa revealed differences in the direction of base substitutions (F(2) = 5,682, *p* < 0.001; multifactor ANOVA). Moreover, there are significantly more base substitutions at G sites associated with 8-oxo-dG damage in the FEL compared to the SEL (Fig 6H; *p* < 0.001; Tukey honest significance differences post hoc test). These analyses reveal that FEL genomes have higher proportions of G site substitutions associated with the mutational signature of a common endogenous mutagen.

#### *Hanseniaspora* FEL yeasts have a greater genomic signature of UV damage

UV damage can result in C → T substitutions at CC sites and CC → TT double substitutions [20,65]. Although both the FEL and SEL have lost *PHR1*, a gene encoding a DNA photolyase that repairs pyrimidine dimers, the FEL has lost additional genes in other pathways that can repair UV damage (e.g., *POL32* in the excision repair pathways). We hypothesized the FEL would have a greater signature of UV damage due to these gene losses. We found significantly greater number of single and double substitutions in CC sites indicative of UV damage in the FEL compared to the SEL (Fig 6I; *p* < 0.001 for both tests; Wilcoxon rank–sum test).

Lastly, we examined whether all of these mutations were associated with more radical amino-acid changes in the FEL compared to the SEL using two measures of amino-acid change: Sneath’s index [66] and Epstein’s coefficient of difference [67]. For both measures, we observed significantly more radical amino-acid substitutions in the FEL compared to the SEL (S11 Fig; *p* < 0.001; Wilcoxon rank–sum test for both metrics). Altogether, these analyses reveal greater DNA sequence instability in the FEL compared to the SEL, which is also associated with more radical amino-acid substitutions.

## Discussion

Species in the genus *Hanseniaspora* exhibit the longest branches among budding yeasts, and their genomes have some of the lowest numbers of genes, lowest GC contents, and smallest assembly sizes in the subphylum (Fig 1, S4 Fig) [34–36]. Through the analysis of the genomes of nearly every known *Hanseniaspora* species, this study presents multiple lines of evidence suggesting that one lineage of *Hanseniaspora*, which we have named the FEL, is a lineage of long-term, hypermutator species that have undergone extensive gene loss (Figs 1–4 as well as S2, S5, S7 and S8 Figs).

Evolution by gene loss is gaining increasing attention as a major mode of genome evolution [35,68] and is mainly possible because of the dispensability of the majority of genes. E.g., 90% of *E*. *coli* [69], 80% of *S*. *cerevisiae* [70], and 73% of *Candida albicans* [71] genes are dispensable in laboratory conditions. The loss of dispensable genes can be selected for [72] and is common in lineages of obligate parasites or symbionts such as in the microsporidia, intracellular fungi that have lost key metabolic pathways such as amino-acid biosynthesis pathways [73,74], and myxozoa, a group of cnidarian obligate parasites that infect vertebrates and invertebrates [75]. Similar losses are also increasingly appreciated in free-living organisms, such as the budding yeasts [this study; 34,35,76–78] and animals [68]. E.g., the loss of *SUC2*, a gene known to enable sucrose utilization [47], in the FEL reflects the inability of species in the FEL to grow on sucrose, while its presence in the SEL reflects its species’ ability to grow on sucrose (Fig 2).

However, *Hanseniaspora* species have experienced not just the typically observed losses of metabolic genes (Fig 2A and 2B) but, more strikingly, the atypical loss of dozens of cell-cycle and DNA damage, response, and repair genes (Figs 3 and 4). Losses of cell-cycle genes are extremely rare [11], and most such losses are known in the context of cancers [79]. Losses of individual or a few DNA repair genes have also been observed in individual hypermutator fungal isolates [6–8]. In contrast, the *Hanseniaspora* losses of cell-cycle and DNA repair genes are not only unprecedented in terms of the numbers of genes lost and their striking impact on genome sequence evolution but also in terms of the evolutionary longevity of the lineage.

### Lost checkpoint processes are associated with fast growth and bipolar budding

*Hanseniaspora* species lost numerous components of the cell cycle (Fig 3), such as *WHI5*, which causes accelerated G1/S transitions in knock-out *S*. *cerevisiae* strains [12,51], as well as components of APC (i.e., *CDC26* and *MND2*), which may accelerate the transition to anaphase [13]. These and other cell-cycle–gene losses are suggestive of rapid cell division and growth and consistent with the known ability of *Hanseniaspora* yeast for rapid growth in the wine fermentation environment [41].

One of the distinguishing characteristics of the *Hanseniaspora* cell cycle is bipolar budding, which is known only in the genera *Wickerhamia* (Debaryomycetaceae) and *Nadsonia* (Dipodascaceae), as well as in *Hanseniaspora* and its sister genus *Saccharomycodes* (both in the family Saccharomycodaceae) [45,80]. These three lineages are distantly related to one another on the budding yeast phylogeny [35], so bipolar budding likely evolved three times independently in Saccharomycotina, including in the last common ancestor of *Hanseniaspora* and *Saccharomycodes*. Currently, there is only one genome available for *Saccharomycodes* [80], making robust inferences of ancestral states challenging. Interestingly, examination of cell-cycle–gene presence and absence in the only representative genome from the genus, *Saccharomycodes ludwigii* [80], reveals that *CDC26*, Pho85 CycLin 1 (*PCL1*), Precocious Dissociation of Sisters 1 (*PDS1*), *RFX1*, Substrate/Subunit Inhibitor of Cyclin-dependent protein kinase 1 (*SIC1*), SPOrulation 12 (*SPO12*), and *WHI5* are absent (S6 File), most of which are either absent from all *Hanseniaspora* (i.e., *CDC26*, *RFX1*, *SPO12*, and *WHI5*) or just from the FEL (i.e., *PDS1* and *SIC1*). This evidence raises the hypothesis that bipolar budding is linked to the dysregulation of cell-cycle processes because of the absence of cell-cycle genes and in particular cell-cycle checkpoints (Fig 3).

### Some gene losses may be compensatory

Deletion of many of the genes associated with DNA maintenance that have been lost in *Hanseniaspora* leads to dramatic increases of mutation rates and gross genome instability [12,13,20], raising the question of how these gene losses were tolerated in the first place. Examination of the functions of the genes lost in *Hanseniaspora* suggests that at least some of these gene losses may have been compensatory. E.g., *POL4* knock-out strains of *S*. *cerevisiae* can be rescued by the deletion of Yeast KU protein 70 (*YKU70*) [81], both of which were lost in the FEL. Similarly, the loss of genes responsible for key cell-cycle functions (e.g., kinetochore functionality and chromosome segregation) appears to have co-occurred with the loss of checkpoint genes responsible for delaying the cell cycle if its functions fail to complete, which may have allowed *Hanseniaspora* cells to bypass otherwise detrimental cell-cycle arrest. Specifically, *MAD1* and *MAD2*, which help delay anaphase when kinetochores are unattached [14]; the 10-gene DASH complex, which participates in spindle attachment, stability, and chromosome segregation [82]; and the 4-gene MIND complex, which is required for kinetochore biorientation and accurate chromosome segregation [83], were all lost in the FEL.

Lastly, the telomere-capping protein *CDC13* was lost in the FEL but is essential not only in *S*. *cerevisiae* but also in mammalian cells. However, additional losses in DNA-damage–response genes (i.e., Slow Growth Suppressor 1 [*SGS1*], EXOnuclease 1 [*EXO1*], and *RAD9*) can allow yeast cells to survive in the absence of *CDC13* [84]. In addition to *CDC13*, the FEL has also lost the checkpoint protein *RAD9* and other genes in the DNA-damage–checkpoint pathway, including Mediator of the Replication Checkpoint 1 (*MRC1*) and *MEC3*. We hypothesize that the loss of *CDC13* was compensated by losses in the DNA-damage–response pathway, as has been observed in *S*. *cerevisiae* [84].

### Long-term hypermutation and the subsequent slowing of sequence evolution

Estimates of the substitution rate ratio ω suggest the FEL and SEL, albeit to a much lower degree in the latter, underwent a burst of accelerated sequence evolution in their stem lineages, followed by a reduction in the pace of sequence evolution (Fig 5). This pattern is consistent with theoretical predictions that selection against mutator phenotypes will reduce the overall rate of sequence evolution [27], as well as with evidence from experimental evolution of hypermutator lines of *S*. *cerevisiae* that showed that their mutation rates were quickly reduced [33]. Although we do not know the catalyst for this burst of sequence evolution, hypermutators may be favored in maladapted populations or in conditions in which environmental parameters frequently change [27,33]. While the environment occupied by the *Hanseniaspora* last common ancestor is unknown, it is plausible that environmental instability or other stressors favored hypermutators in *Hanseniaspora*. Extant *Hanseniaspora* species are well known to be associated with the grape environment [40,85,86]. Interestingly, grapes appear to have originated [87] around the same time window that *Hanseniaspora* did (Fig 1B), leading us to speculate that the evolutionary trigger of *Hanseniaspora* hypermutation could have been adaptation to the grape environment.

### Losses of DNA repair genes are reflected in patterns of sequence evolution

Although the relationship between genotype and phenotype is complex, the loss of genes involved in DNA repair can have predictable outcomes on patterns of sequence evolution in genomes. In the case of the observed losses of DNA repair genes in *Hanseniaspora*, the mutational signatures of this loss and the consequent hypermutation can be both general (i.e., the sum total of many gene losses), as well as specific (i.e., can be putatively linked to the losses of specific genes or pathways). Arguably the most notable general mutational signature is that *Hanseniaspora* genome sequence evolution is largely driven by random (i.e., neutral) mutagenic processes with a strong AT bias. E.g., whereas the transition/transversion ratios of eukaryotic genomes are typically within the 1.7 and 4 range [88–91], *Hanseniaspora* ratios are approximately 0.66–0.75 (Fig 6C), which are values on par with estimates of transition/transversion caused by neutral mutations alone (e.g., 0.6–0.95 in *S*. *cerevisiae* [88,92], 0.92 in *E*. *coli* [93], 0.98 in *Drosophila melanogaster* [94], and 1.70 in humans [95]). Similarly, base substitutions across *Hanseniaspora* genomes are strongly AT biased, especially in the FEL (Fig 6), an observation consistent with the general AT bias of mutations observed in diverse organisms, including numerous bacteria [96], *Drosophila* fruit flies [94], *S*. *cerevisiae* [88], and humans [95].

In addition to these general mutational signatures, examination of *Hanseniaspora* sequence evolution also reveals mutational signatures that can be linked to the loss of specific DNA repair genes. E.g., we found a higher proportion of base substitutions associated with the most abundant oxidatively damaged base—8-oxo-dG, which causes G → T or C → A transversions [26]—in the FEL compared to the SEL, which reflects specific gene losses. Specifically, *Hanseniaspora* yeasts have lost *PCD1*, which encodes a diphosphatase that contributes to the removal of 8-oxo-dGTP [24] and thereby reduces the chance of misincorporating this damaged base. Once 8-oxo-dG damage has occurred, it is primarily repaired by the base-excision repair pathway [26]. Notably, the FEL has lost a key component of the base-excision repair pathway, a DNA polymerase δ subunit, encoded by *POL32*, which aids in filling the gap after excision [97]. Accordingly, the proportion of G|C sites with substitutions indicative of 8-oxo-dG damage (i.e., G → T or C → A transversions) is significantly greater in the FEL compared to the SEL (Fig 5H). Similarly, the numbers of dinucleotide substitutions of CC → TT associated with UV-induced pyrimindine dimers [98] are higher in the FEL compared to the SEL (Fig 5I) due to the loss of PHR1 and other alternative pathways that repair UV damage [20,65].

Our analyses provide the first, to our knowledge, major effort to characterize the genome function and evolution of the enigmatic genus *Hanseniaspora*. Our analyses focus on genomic differences between two lineages and identify major and extensive losses of genes associated with metabolism, cell-cycle, and DNA repair processes. These extensive losses and the concomitant acceleration of evolutionary rate mean that levels of amino-acid sequence divergence within each of the two *Hanseniaspora* lineages alone, but especially within the FEL, are similar to those observed within plant classes and animal subphyla (S12 Fig). These discoveries set the stage for further examination of intralineage or intraspecies variation in genomic features and content. More interestingly, our analyses lay the foundation for fundamental molecular and evolutionary investigations among *Hanseniaspora*, such as potential novel rewiring of cell-cycle and DNA repair processes.

## Methods

### DNA sequencing

For each species, genomic DNA (gDNA) was isolated using a two-step phenol:chloroform extraction previously described to remove additional proteins from the gDNA [35]. The gDNA was sonicated and ligated to Illumina sequencing adaptors as previously described [99], and the libraries were submitted for paired-end sequencing (2 × 250) on an Illumina HiSeq 2500 instrument (Illumina, San Diego, CA, USA).

### Phenotyping

We qualitatively measured growth of species on five carbon sources (maltose, raffinose, sucrose, melezitose, and galactose) as previously described in [35]. We used a minimal media base with ammonium sulfate, and all carbon sources were at a 2% concentration. Yeast were initially grown in YPD and transferred to carbon treatments. Species were visually scored for growth for about a week on each carbon source in three independent replicates over multiple days. A species was considered to utilize a carbon source if it showed growth across ≥50% of biological replicates. Growth data for *H*. *gamundiae* were obtained from Čadež and colleagues [42].

### Genome assembly and annotation

To generate de novo genome assemblies, we used paired-end DNA sequence reads as input to iWGS, version 1.1 [100], a pipeline that uses multiple assemblers and identifies the “best” assembly according to largest genome size and N50 (i.e., the shortest contig length among the set of the longest contigs that account for 50% of the genome assembly’s length) [101] as described in [35]. More specifically, sequenced reads were first quality trimmed, and adapter sequences were removed using Trimmomatic, version 0.33 [102] and Lighter, version 1.1.1 [103]. Subsequently, KmerGenie, version 1.6982 [104] was used to determine the optimal *k*-mer length for each genome individually. Thereafter, six de novo assembly tools (i.e., ABYSS, version 1.5.2 [105]; DISCOVAR, release 51885 [106]; MASURCA, version 2.3.2 [107]; SGA, version 0.10.13 [108]; SOAPdenovo2, version 2.04 [109]; and SPADES, version 3.7.0 [110]) were used to generate genome assemblies from the processed reads. Using QUAST, version 4.4 [111], the best assembly was chosen according to the assembly that provided the largest genome size and best N50.

Annotations for eight of the *Hanseniaspora* genomes (i.e., *H*. *clermontiae*, *H*. *osmophila* CBS 313, *H*. *pseudoguilliermondii*, *H*. *singularis*, *H*. *uvarum* DSM2768, *H*. *valbyensis*, *H*. *vineae* T02 19AF, and *K*. *hatyaiensis*) and the four outgroup species (i.e., *Cyberlindnera jadinii*, *Kluyveromyces marxianus*, *S*. *cerevisiae*, and *Wickerhamomyces anomalus*) were generated in a recent comparative genomic study of the budding yeast subphylum [35]. The other 11 *Hanseniaspora* genomes examined here were annotated by following the same protocol as in [35].

In brief, the genomes were annotated using the MAKER pipeline, version 2.31.8 [112]. The homology evidence used for MAKER consists of fungal protein sequences in the SwissProt database (release 2016_11) and annotated protein sequences of select yeast species from Mycocosm [113], a web portal developed by the US Department of Energy Joint Genome Institute for fungal genomic analyses. Three ab initio gene predictors were used with the MAKER pipeline, including GeneMark-ES, version 4.32 [114]; SNAP, version 2013-11-29 [115]; and AUGUSTUS, version 3.2.2 [116], each of which was trained for each individual genome. GeneMark-ES was self-trained on the repeat-masked genome sequence with the fungal-specific option (“–fugus”), while SNAP and AUGUSTUS were trained through three iterative MAKER runs. Once all three ab initio predictors were trained, they were used together with homology evidence to conduct a final MAKER analysis in which all gene models were reported (“keep_preds” set to 1), and these comprise the final set of annotations for the genome.

### Data acquisition

All publicly available *Hanseniaspora* genomes, including multiple strains from a single species, were downloaded from NCBI (https://www.ncbi.nlm.nih.gov/; S1 File). These species and strains include *H*. *guilliermondii* UTAD222 [85], *H*. *opuntiae* AWRI3578, *H*. *osmophila* AWRI3579, *H*. *uvarum* AWRI3580 [117], *H*. *uvarum* 34–9, *H*. *vineae* T02 19AF [118], *H*. *valbyensis* NRRL Y-1626 [34], and *H*. *gamundiae* [42]. We also included *S*. *cerevisiae* S288C, *K*. *marxianus* DMKU3-1042, *W*. *anomalus* NRRL Y-366-8, and *C*. *jadinii* NRRL Y-1542, four representative budding yeast species that are all outside the genus *Hanseniaspora* [35], which we used as outgroups. Together with publicly available genomes, our sampling of *Hanseniaspora* encompasses all known species in the genus (or its anamorphic counterpart, *Kloeckera*), except *Hanseniaspora lindneri*, which likely belongs to the FEL based on a four-locus phylogenetic study [119], and *Hanseniaspora taiwanica*, which likely belongs to the SEL based on neighbor-joining analyses of the LSU rRNA gene sequence [56].

### Assembly assessment and identification of orthologs

To determine genome assembly completeness, we calculated contig N50 [101] and assessed gene-content completeness using multiple databases of curated orthologs from BUSCO, version 3 [120]. More specifically, we determined gene-content completeness using orthologous sets of genes constructed from sets of genomes representing multiple taxonomic levels, including Eukaryota (superkingdom; 100 species; 303 BUSCOs), Fungi (kingdom; 85 species; 290 BUSCOs), Dikarya (subkingdom; 75 species; 1,312 BUSCOs), Ascomycota (phylum; 75 species; 1,315 BUSCOs), Saccharomyceta (no rank; 70 species; 1,759 BUSCOs), and Saccharomycetales (order; 30 species; 1,711 BUSCOs).

Genomes sequenced in the present project were sequenced at an average depth of 63.49 ± 52.57 (S1 File). Among all *Hanseniaspora*, the average scaffold N50 was 269.03 ± 385.28 kb, the average total number of scaffolds was 980.36 ± 835.20 (398.32 ± 397.97 when imposing a 1 kb scaffold filter), and the average genome assembly size was 10.13 ± 1.38 Mb (9.93 ± 1.35 Mb when imposing a 1 kb scaffold filter). Notably, the genome assemblies and gene annotations created in the present project were comparable to publicly available ones. E.g., the genome size of publicly available *H*. *vineae* T02 19AF is 11.38 Mb with 4,661 genes, while our assembly of *H*. *vineae* NRRL Y-1626 was 11.15 Mb with 5,193 genes.

We found that our assemblies were of comparable quality to those from publicly available genomes. E.g., *H*. *uvarum* NRRL Y-1614 (N50 = 267.64 kb; genome size = 8.82 Mb; number of scaffolds = 258; gene number = 4,227), which was sequenced in the present study, and *H*. *uvarum* AWRI3580 (N50 = 1,289.09 kb; genome size = 8.81 Mb; number of scaffolds = 18; gene number = 4,061), which is publicly available [117], had similar single-copy BUSCO genes present in the highest and lowest orthoDB [43] taxonomic ranks (Eukaryota and Saccharomycetales, respectively). Specifically, *H*. *uvarum* NRRL Y-1614 and *H*. *uvarum* AWRI3580 had 80.20% (243/303) and 79.87% (242/303) of universally single-copy orthologs in Eukaryota present in each genome, respectively, and 52.31% (895/1,711) and 51.49% (881/1,711) of universally single-copy orthologs in Saccharomycetales present in each genome, respectively.

To identify single-copy OGs among all protein coding sequences for all 29 taxa, we used OrthoMCL, version 1.4 [121]. OrthoMCL clusters genes into OGs using a Markov clustering algorithm [122; https://micans.org/mcl/] from gene similarity information acquired from a blastp “all-vs-all” using NCBI’s Blast+, version 2.3.0 (S2 Fig; [123]) and the proteomes of species of interest as input. The key parameters used in blastp “all-vs-all” were e-value = 1 × 10^−10^, percent identity cutoff = 30%, percent match cutoff = 70%, and a maximum weight value = 180. To conservatively identify OGs, we used a strict OrthoMCL inflation parameter of 4.

To identify additional OGs suitable for use in phylogenomic and molecular sequence analyses, we identified the single best putatively orthologous gene from OGs with full species representation and a maximum of two species with multiple copies using PhyloTreePruner, version 1.0 [124]. To do so, we first aligned and trimmed sequences in 1,143 OGs out of a total of 11,877 that fit the criterion of full representation and a maximum of two species with duplicate sequences. More specifically, we used Mafft, version 7.294b [125], with the Blosum62 matrix of substitutions [126], a gap penalty of 1.0, 1,000 maximum iterations, the “genafpair” parameter, and trimAl, version 1.4 [127], with the “automated1” parameter to align and trim individual sequences, respectively. The resulting OG multiple sequence alignments were then used to infer gene phylogenies using FastTree, version 2.1.9 [128], with 4 and 2 rounds of subtree–prune–regraft and optimization of all 5 branches at nearest-neighbor interchanges, respectively, as well as the “slownni” parameter to refine the inferred topology. Internal branches with support lower than 0.9 Shimodaira–Hasegawa-like support implemented in FastTree [128] were collapsed using PhyloTreePruner, version 1.0 [124], and the longest sequence for species with multiple sequences per OG were retained, resulting a robust set of OGs with every taxon being represented by a single sequence. OGs were realigned (Mafft) and trimmed (trimAl) using the same parameters as above.

### Phylogenomic analyses

To infer the *Hanseniaspora* phylogeny, we performed phylogenetic inference using maximum likelihood [129] with concatenation [130,131] and coalescence [132] approaches. To determine the best-fit phylogenetic model for concatenation and generate single-gene trees for coalescence, we constructed trees per single-copy OG using RAxML, version 8.2.8. [133], in which each topology was determined using 5 starting trees. Single-gene trees that did not recover all outgroup species as the earliest diverging taxa when serially rooted on outgroup taxa were discarded. Individual OG alignments or trees were used for species tree estimation with RAxML (i.e., concatenation) using the LG [134] model of substitution, which is the most commonly supported model of substitution (874/1,034; 84.53% genes), or Astral-II, version 4.10.12 (i.e., coalescence) [135]. Branch support for the concatenation and coalescence phylogenies was determined using 100 rapid bootstrap replicates [136] and local posterior support [132], respectively.

Several previous phylogenomic studies have shown that the internal branches preceding the *Hanseniaspora* FEL and SEL are long [34,36]. To examine whether the relationship between the length of the internal branch preceding the FEL and the length of the internal branch preceding the SEL was consistent across genes in our phylogeny, we used Newick Utilities, version 1.6 [137] to remove the 88 single-gene trees in which either lineage was not recovered as monophyletic and calculated their difference for the remaining 946 genes.

### Estimating divergence times

To estimate divergence times among the 25 *Hanseniaspora* genomes, we used the Bayesian method MCMCTree in paml, version 4.9 [138] and the concatenated 1,034-gene matrix. The input tree was derived from the concatenation-based ML analysis under a single LG + G4 [134] model (Fig 1A). The in-group root (i.e., the split between the FEL and the SEL) age was set between 0.756 and 1.177 time units (1 time unit = 100 mya), which was adopted from a recent study [35].

To infer the *Hanseniaspora* time tree, we first estimated branch lengths under a single LG + G4 [134] model with codeml in the paml, version 4.9 [138] package and obtained a rough mean of the overall mutation rate. Next, we applied the approximate likelihood method [139,140] to estimate the gradient vector and Hessian matrix with Taylor expansion (option usedata = 3). Last, we assigned (i) the gamma-Dirichlet prior for the overall substitution rate (option rgene_gamma) as G(1, 1.55), with a mean of 0.64; (ii) the gamma-Dirichlet prior for the rate-drift parameter (option sigma2 gamma) as G(1, 10); and (iii) the parameters for the birth–death sampling process with birth and death rates λ = μ = 1 and sampling fraction ρ = 0. We employed the independent-rate model (option clock = 2) to account for the rate variation across different lineages and used soft bounds (left and right tail probabilities = 0.025) to set minimum and maximum values for the in-group root mentioned above. The MCMC run was first run for 1,000,000 iterations as burn-in and then sampled every 1,000 iterations until a total of 30,000 samples was collected. Two separate MCMC runs were compared for convergence, and similar results were observed.

### Gene presence and absence analysis

To determine the presence and absence of genes in *Hanseniaspora* genomes, we built HMMs for each gene present in *S*. *cerevisiae* and used the resulting HMM profile to search for the corresponding homolog in each *Hanseniaspora* genome, as well as outgroup taxa. More specifically, for each of the 5,917 verified open reading frames from *S*. *cerevisiae* [141] (downloaded October 2018 from the *Saccharomyces* Genome Database), we searched for putative homologs in NCBI’s Reference Sequence Database for Fungi (downloaded June 2018) using NCBI’s Blast+, version 2.3.0 [142] blastp function and an e-value cutoff of 1 × 10^−3^, as recommended for homology searches [143]. We used the top 100 hits for the gene of interest and aligned them using Mafft, version 7.294b [125], with the same parameters described above. The resulting gene alignment was then used to create an HMM profile for the gene using the hmmbuild function in HMMER, version 3.1b2 [144]. The resulting HMM profile was then used to search for each individual gene in each *Hanseniaspora* genome and outgroup taxa using the hmmsearch function with an expectation value cutoff of 0.01 and a score cutoff of 50. This analysis was done for the 5,735 genes with multiple blast hits, allowing for the creation of an HMM profile. To evaluate the validity of constructed HMMs, we examined their ability to recall genes in *S*. *cerevisiae* and found that we recovered all nuclear genes.

To determine whether any functional categories were over- or under-represented among genes present or absent among *Hanseniaspora* species, we conducted GO [145] enrichment analyses using GOATOOLS, version 0.7.9 [146]. We used a background of all *S*. *cerevisiae* genes and a *p*-value cutoff of 0.05 after multiple-test correction using the Holm method [147]. Plotting gene presence and absence among pathways was done by examining depicted pathways available through the KEGG project [148] and the *Saccharomyces* Genome Database [141].

We examined the validity of the gene presence and absence pipeline by examining under-represented terms and the presence or absence of essential genes in *S*. *cerevisiae* [149]. We hypothesized that under-represented GO terms will be associated with basic molecular processes and that essential genes will be under-represented among the set of absent genes. In agreement with these expectations, GO terms associated with basic biological processes and essential *S*. *cerevisiae* genes are under-represented among genes that are absent across *Hanseniaspora* genomes. E.g., among all genes absent in the FEL and SEL, the molecular functions BASE PAIRING, GO:0000496 (*p* < 0.001); GTP BINDING, GO:0005525 (*p* < 0.001); and ATPASE ACTIVITY, COUPLED TO MOVEMENT OF SUBSTANCES, GO:0043492 (*p* < 0.001) are significantly under-represented (S4 File). Similarly, *S*. *cerevisiae* essential genes are significantly under-represented (*p* < 0.001; Fischer’s exact test for both lineages) among lost genes, with 134 and 23 *S*. *cerevisiae* essential genes having been lost from the FEL and SEL genomes, respectively (lists of essential *S*. *cerevisiae* genes absent among *Hanseniaspora* genomes are available through figshare 10.6084/m9.figshare.7670756).

### Ploidy estimation

To determine ploidy, we leveraged base frequency distributions at variable sites by mapping each genome’s reads to its assembly. This approach is widely employed to determine ploidy from next-generation sequencing data and has been implemented in several pieces of software [150–152] and studies [153,154]. In short, examination of base frequency distributions between a frequency of 20 and 80 can provide insight into ploidy status. More specifically, haploid genomes lack biallelic sites, so their base frequency distributions will peak at high and low base frequencies and be depleted in positions with base frequencies near 50 (or a “smiley pattern”); diploid genomes typically have two alleles for a locus and are expected to exhibit a unimodal distribution centered around a base frequency of 50; finally, triploid genomes typically have one allele on one chromosome and the other allele in the other two chromosomes and are expected to exhibit a bimodal distribution centered around base frequencies of 33 and 66. Note that this approach assumes that there is a sufficient amount of heterozygosity in the genome and that ploidy changes may be go undetected in genomes lacking heterozygosity. To ensure high-quality read mapping, we first quality-trimmed reads suing Trimmomatic, version 0.36 [102], using the parameters leading:10, trailing:10, slidingwindow:4:20, and minlen:50. Reads were subsequently mapped to their respective genome using bowtie2, version 1.1.2 [155], with the “sensitive” parameter, and we converted the resulting file to a sorted bam format using SAMtools, version 1.3.1 [156]. We next used nQuire [151], which extracts base frequency information at segregating sites with a minimum frequency of 0.2. Prior to visualization, we removed background noise by utilizing the Gaussian mixture model with uniform noise component [151].

### Molecular evolution and mutation analysis

#### Molecular sequence rate analysis along the phylogeny

To determine the rate of sequence evolution over the course of *Hanseniaspora* evolution, we examined variation in the rate of dN to the rate of synonymous dS substitutions (dN/dS or ω) across the species phylogeny. We first obtained codon-based alignments of the protein sequences used during phylogenomic inference by threading nucleotides on top of the amino-acid sequence using pal2nal, version 14 [157] and calculated ω values under the different hypotheses using the codeml module in paml, version 4.9 [138]. For each gene tested, we set the null hypothesis (H_O_) where all internal branches exhibit the same ω (model = 0) and compared it to four different alternative hypotheses. Under the H_FEL–SEL branch_ hypothesis, the branches immediately preceding the FEL and SEL were assumed to exhibit distinct ω values from the background (model = 2) (Fig 5Bi). Under the H_FEL_ hypothesis, the branch immediately preceding the FEL was assumed to have a distinct ω value, all FEL crown branches were assumed to have their own collective ω value, and all background branches were assumed to have their own collective ω value (model = 2) (Fig 5Ci). The H_SEL_ hypothesis assumed the branch preceding the lineage had its own ω value, all SEL crown branches had their own collective ω value, and all background branches were assumed to have their own collective ω value (model = 2) (Fig 5Di). Lastly, the H_FEL–SEL crown_ hypothesis assumed that all FEL crown branches had their own collective ω value, all SEL crown branches had their own collective ω value, and the rest of the branches were assumed to have their own collective ω value (model = 2) (Fig 5Ei). To determine whether each of the alternative hypotheses was significantly different from the null hypothesis, we used the LRT (α = 0.01). A few genes could not be analyzed because of fatal interruptions or errors during use in paml, version 4.9 [138], which have been reported by other users [158]; these genes were removed from the analysis. Thus, this analysis was conducted for 989 genes for three tests (H_FEL–SEL branch_, H_FEL_, and H_SEL_ hypotheses) and 983 genes for one test (H_FEL–SEL crown_ hypothesis).

#### Examination of mutational signatures

To conservatively identify base substitutions, insertions, and deletions found in taxa in the FEL or SEL, we examined the status of each nucleotide at each position in codon-based and amino-acid–based OG alignments. We examined base substitutions, insertions, and deletions at sites that are conserved in the outgroup (i.e., all outgroup taxa have the same character state for a given position in an alignment). For base substitutions, we determined if the nucleotide or amino-acid residue in a given *Hanseniaspora* species differed from the conserved outgroup nucleotide or amino-acid residue at the same position. To measure whether amino-acid substitutions in each lineage were conservative or radical (i.e., a substitution to a similar amino-acid residue versus a substitution to an amino-acid residue with different properties), we used Sneath’s index of dissimilarity, which considers 134 categories of biological activity and chemical change to quantify dissimilarity of amino-acid substitutions, and Epstein’s coefficient of difference, which considers differences in polarity and size of amino acids to quantify dissimilarity. Notably, Sneath’s index is symmetric (i.e., isoleucine to leucine is equivalent to leucine to isoleucine), whereas Epstein’s coefficient is not (i.e., isoleucine to leucine is not equivalent to leucine to isoleucine). For indels, we used a sliding window approach with a step size of one nucleotide. We considered positions in which a nucleotide was present in all outgroup taxa but a gap was present in *Hanseniaspora* as deletions and positions in which a gap was present in all outgroup taxa and a nucleotide was present in *Hanseniaspora* species as insertions. Analyses were conducted using custom Python, version 3.5.2 (https://www.python.org/) scripts, which use the BioPython, version 1.70 [159] and numpy, version 1.13.1 [160] modules.

We discovered that all *Hanseniaspora* species lack the *PHR1* gene, which is associated with the repair of UV radiation damage, but the FEL has lost additional genes that participate in other pathways that can repair UV damage such as the base-excision and nucleotide-excision repair pathway [20,65]. UV radiation induces high levels of C → T substitutions at CC sites and, more rarely, double substitutions of CC → TT [98,161]. To examine signatures of UV radiation damage across *Hanseniaspora*, we examined the number of C → T substitutions at CC sites (or G → A substitutions at GG sites) as well as the less frequent CC → TT (or GG → AA) double substitutions.

## Supporting information

S1 Fig Phylogenomics method pipeline. Using 25 *Hanseniaspora* proteomes and the proteomes of 4 outgroup taxa, we identified 11,877 orthologous groups of genes. For 1,143 orthologous groups, ≥90% of taxa were represented by a single sequence, while the others had two sequences (i.e., putative paralogs). The sequences of the 1,143 orthologous groups were individually aligned, trimmed, had their evolutionary history inferred, and paralogs were trimmed based on tree topology. Using the resulting 1,142 OGs with paralogs trimmed, sequences were realigned, trimmed, and had their evolutionary history inferred. Orthologous groups where the outgroup taxa were not recovered as the sister clade to the genus *Hanseniaspora* were removed, reducing the set to 1,034 orthologous groups. Among these 1,034 orthologous groups of genes, a concatenated 1,034-gene matrix was constructed and used for reconstructing evolutionary history. Similarly, evolutionary history was inferred using coalescence of the 1,034 orthologous group single-gene phylogenies. OG, orthologous gene.
(TIF)

S2 Fig Concatenation and coalescence produce nearly identical and well-supported phylogenies that support two distinct *Hanseniaspora* lineages. (Left) Concatenation provides support for a lineage with a long stem branch, which we term the FEL, and another lineage with a much shorter stem branch, which we term the SEL. (Right) Coalescence supports monophyly of the FEL and SEL. Minor discrepancies are observed between the topologies. Only the values for bipartitions without full support are shown. Support for concatenation and coalescence was determined using 100 rapid bootstrap replicates and local posterior support, respectively. figshare: https://doi.org/10.6084/m9.figshare.7670756.v2. FEL, faster-evolving lineage; SEL, slower-evolving lineage.
(TIF)

S3 Fig Internode key to accompany divergence time estimate file per internode. Internode identifiers for time tree analysis are shown in Fig 1B. Associated mean divergence time and credible intervals can be found in the S2 File.
(TIF)

S4 Fig *Hanseniaspora* have among the smallest genome sizes, lowest number of genes, and lowest GC content percentages in the budding yeast subphylum Saccharomycotina. (A) The genus *Hanseniaspora* (family Saccharomycodaceae) includes the smallest budding yeast genome. Average genome sizes in the FEL, SEL, and the Saccharomycotina are 9.71 ± 1.32 Mb (min: 8.10; max: 14.05), 10.99 ± 1.66 Mb (min: 7.34; max: 12.17), 12.80 ± 3.20 Mb (min: 7.34; max: 25.83), respectively. (B) The genus *Hanseniaspora* includes the budding yeast genome with the fewest genes. Average number of genes per genome in the FEL, SEL, and Saccharomycotina are 4,707.89 ± 633.56 (min: 3,923; max: 6,380), 4,932.43 ± 289.71 (min: 4,624; max: 5,349), and 5,657.66 ± 1,044.78 (min: 3,923; max: 12,786), respectively. (C) The genus *Hanseniaspora* has among the lowest GC content values in budding yeast genomes. GC content values in the FEL, SEL, and Saccharomycotina are 33.10 ± 3.53% (min: 26.32; max: 37.17), 37.28 ± 2.05% (min: 34.82; max: 39.93), and 40.30 ± 5.71% (min: 25.2; max: 53.98), respectively. Families of Saccharomycotina are depicted on the *y*-axis. Median values are depicted with a line, and dashed lines indicate plus or minus one standard deviation from the median. To the right of each figure, boxplots depict the median and standard deviations of each grouping. The gray represents all of Saccharomycotina. Blue represents the SEL, and orange represents the FEL. figshare: https://doi.org/10.6084/m9.figshare.7670756.v2. FEL, faster-evolving lineage; GC, Guanine–Cytosine; max, maximum; min, minimum; SEL, slower-evolving lineage.
(TIF)

S5 Fig BUSCO analyses reveal extensive gene absence across various taxonomic ranks. BUSCO [120] analyses of *Hanseniaspora* proteomes using the Eukaryota (n_BUSCOs_ = 303), Fungi (n_BUSCOs_ = 290), Dikarya (n_BUSCOs_ = 1,312), Ascomycota (n_BUSCOs_ = 1,315), Saccharomyceta (n_BUSCOs_ = 1,759), and Saccharomycetales (n_BUSCOs_ = 1,711) orthoDB databases revealed that very large numbers of BUSCO genes are absent from *Hanseniaspora* genomes and from FEL genomes in particular. figshare: https://doi.org/10.6084/m9.figshare.7670756.v2. BUSCO, Benchmarking Universal Single-Copy Orthologs; FEL, faster-evolving lineage.
(TIF)

S6 Fig A liberal targeted gene-searching pipeline and the number of genes absent in at least two-thirds of FEL and SEL taxa. (A) A FASTA file for gene *X*, where gene *X* is the FASTA entry of a verified ORF in the *S*. *cerevisiae* proteome, was used as a query to search for putative homologs in the Fungal reference sequence (refseq) database. The top 100 putative homologs were subsequently aligned. From the alignment, an HMM was made. Using the HMM, gene *X* was searched for in the genome of each species from the FEL, SEL, and outgroup individually using a liberal e-value cutoff of 0.01 and a score of >50. This pipeline yields presence and absence information of gene *X* among FEL, SEL, and outgroup taxa. This method was subsequently applied to all verified ORFs in the *S*. *cerevisiae* proteome. FEL, faster-evolving lineage; HMM, Hidden Markov Model; ORF, open reading frame; refseq, reference sequence; SEL, slower-evolving lineage.
(TIF)

S7 Fig Gene presence and absence reveals a putatively diminished gluconeogenesis pathway. Gene presence and absence analysis of genes that participate in the gluconeogenesis (A) and glycolysis (B) pathway reveal the absence of key genes in the gluconeogenesis pathway, suggestive of a diminished capacity for gluconeogenesis. More specifically, *PCK1*, which encodes the enzyme that converts oxaloacetic acid to phosphoenolpyruvate, and *FBP1*, which encodes the enzyme that converts fructose-1,6-bisphosphate to fructose-6-phospbate, are absent from all *Hanseniaspora* species. figshare: https://doi.org/10.6084/m9.figshare.7670756.v2. *FBP1*, Fructose-1,6-BisPhosphatase 1; *PCK1*, Phosphoenolpyruvate CarboxyKinase 1.
(TIF)

S8 Fig Base frequency plots reveal diversity in ploidy of *Hanseniaspora* species. (A) Plots with peaks at high and low base frequencies (or a “smiley pattern”) reflect a lack of biallelic sites, which is suggestive of a haploid genome. The smiley-pattern distributions observed for the genomes of *H*. *occidentalis* var. *occidentalis*, *H*. *uvarum* CBS 314, and *H*. *guilliermondii* CBS 465 suggest these species have haploid genomes. (B) A unimodal distribution centered around a base frequency of 50 is consistent with the presence of two alleles at a given locus and suggestive of a diploid genome. The unimodal distributions centered around a base frequency of 50 suggest *H*. *occidentalis* var. *citrica*, *H*. *osmophila* CBS 313, *H*. *meyeri*, *H*. *clermontiae*, *H*. *nectarophila*, *H*. *thailandica*, *H*. *pseudoguilliermondii*, *H*. *singularis*, and *K*. *hatyaiensis* are diploids. (C) Bimodal distributions centered around base frequencies of 33 and 66 reflect one allele on one chromosome and another allele on the other two chromosomes, which is suggestive of a triploid genome. Bimodal distributions centered around 33 and 66 suggest *H*. *lachancei* and *H*. *jakobsenii* are triploid. (D) Analyses of *H*. *vineae* CBS 2171, *H*. *valbyensis*, *H*. sp. NRRL Y-63759, and *H*. *opuntiae* base frequency distributions were ambiguous. Certain FEL species, such as *H*. *singularis*, *H*. *pseudoguilliermondii*, and *H*. *jakobsenii*, are potentially aneuploid, while evidence of aneuploidy in the SEL is observed only in *H*. *occidentalis* var. *citrica*. figshare: https://doi.org/10.6084/m9.figshare.7670756.v2. CBS, Centraalbureau voor Schimmelcultures; FEL, faster-evolving lineage; NRRL, Northern Regional Research Laboratory; SEL, slower-evolving lineage.
(TIF)

S9 Fig Gene presence and absence related to yeast meiosis. Genes absent in both lineages and the FEL are colored purple and orange, respectively. Dotted lines with arrows indicate indirect links or unknown reactions. Lines with arrows indicate molecular interactions or relations. Circles indicate chemical compounds such as glucose or cAMP. figshare: https://doi.org/10.6084/m9.figshare.7670756.v2. cAMP, cyclic AdenosineMonoPhosphate; FEL, faster-evolving lineage.
(TIF)

S10 Fig Analyses of homopolymers by sequence length, base pair, and type of mutation. Significant differences among the proportion of mutated bases among homopolymers of various lengths were observed (Fig 6). Addition of variables (i.e., sequence type [A|T or C|G] and mutation type [base substitution, insertion, and deletion]) allowed for further determination of what types of mutations caused differences between the FEL and SEL. As shown in Fig 6, we observed significant differences in the numbers of mutations between the FEL and SEL (F = 27.06, *p* < 0.001; multifactor ANOVA) as well as in the type of mutations (F = 1686.70, *p* < 0.001; multifactor ANOVA). A Tukey honest significance differences post hoc test revealed that the proportion of nucleotides that underwent base substitutions was significantly greater than insertions (*p* < 0.001) and deletions (*p* < 0.001). We next focused on significant differences observed between the FEL and SEL when considering all factors. We observed significant differences between the FEL and SEL at A|T and C|G homopolymers with a length of 2 (*p* = 0.009 and *p* < 0.001, respectively), C|G homopolymers of length 3 (*p* < 0.001), and A|T homopolymers of length 5 (*p* < 0.001). figshare: https://doi.org/10.6084/m9.figshare.7670756.v2. FEL, faster-evolving lineage; SEL, slower-evolving lineage.
(TIF)

S11 Fig Metrics reveal more radical amino-acid substitutions in the FEL compared to SEL. Using Sneath’s index and Epstein’s coefficient of difference, the average difference among amino acid substitutions were determined among sites where the outgroup taxa had all the same amino acid. Using either metric, amino-acid substitutions were significantly more radical in the FEL compared to the SEL (*p* < 0.001; Wilcoxon rank–sum test for both metrics). figshare: https://doi.org/10.6084/m9.figshare.7670756.v2. FEL, faster-evolving lineage; SEL, slower-evolving lineage.
(TIF)

S12 Fig Mean protein similarity reveals very high evolutionary genomic diversity in *Hanseniaspora*. Mean protein similarity (measured by amino acid substitutions/site) between species in the FEL (using *H*. *uvarum* as the reference), species in the SEL (using *H*. *vineae* as a reference), Saccharomycetaceae (*S*. *cerevisiae*), animals (human), and plants (thale cress *Arabidopsis thaliana*). For each lineage, mean protein similarity was estimated using a reciprocal best blast hit approach. The mean protein similarity observed in these lineages is roughly on par with genus-level differences within the family Saccharomycetaceae, humans to zebrafish, and thale cress to Japanese rice. Silhouettes were obtained under the Public Domain or Creative Commons license from phylopic.org (human, mouse, zebra finch, frog, zebrafish, and thale cress), from openclipart.org (field mustard, white spruce, wild tomato, Japanese rice; the colors of the field mustard and wild tomato original images were changed to black), or drawn by hand by Jacob L. Steenwyk (all yeasts). FEL, faster-evolving lineage; SEL, slower-evolving lineage.
(TIF)

S1 File Summary table of genomes under study. Information including strain, genome characteristics (e.g., genome size, N50, GC content, number of genes, and other metrics), and source of strains is provided. GC, Guanine–Cytosine.
(XLSX)

S2 File Divergence times for every internode in the *Hanseniaspora* phylogeny. Mean divergences as well as upper and lower confidence intervals are provided for every internode (internal branch length) in the *Hanseniaspora* phylogeny. Internode labels correspond to the labels shown in S4 Fig.
(XLSX)

S3 File Summary of genes absent and present in the FEL and SEL. FEL, faster-evolving lineage; SEL, slower-evolving lineage.
(XLSX)

S4 File GO enrichment analysis of genes absent in *Hanseniaspora*. A summary table that details over- and underenrichment analysis results of genes absent in the *Hanseniaspora* FEL and SEL. GO enrichment results are reported for genes absent in all *Hanseniaspora*, FEL, SEL, and genes uniquely absent in either lineage. FEL, faster-evolving lineage; GO, gene ontology; SEL, slower-evolving lineage.
(XLSX)

S5 File Growth phenotypes across *Hanseniaspora* species and outgroup taxa. Growth phenotypes across eight substrates are depicted here. Among strains examined for a particular growth phenotype, ability to grow was characterized as (1) able to grow on the substrate, (2) unable to grow, or (3) display weak and delayed growth.
(XLSX)

S6 File Summary of cell-cycle–gene presence and absence in *S*. *ludwigii*. A summary table of cell-cycle genes that are either present or absent in the closely related bipolar budding yeast *S*. *ludwigii*.
(XLSX)

## Abbreviations

*ADI*: Acireductone DIoxygenase
APC: Anaphase-Promoting Complex
*ARO*: AROmatic amino-acid requiring
*ASK1*: Associated with Spindles and Kinetochores 1
AWRI3580: Australian Wine Research Institute 3580
*BAT*: Branched-chain Amino-acid Transaminase
CBS 314: Centraalbureau voor Schimmelcultures 314
*CDC13*: Cell Division Cycle 13
CI: credible interval
*DAD*: Death Upon Overproduction 1 And Death Upon Overproduction 1 and MonoPolar Spindle 1 interacting
*DAM1*: Duo1 and MonoPolar Spindle 1
DASH: Dam1 Complex
dN: rate of nonsynonymous substitutions
dS: rate of synonymous substitutions
*DSE2*: Daughter-Specific Expression 2
DSM2768: Dutch State Mines 2768
*DSN1*: Dosage Suppressor of Necessary for Nuclear Function 1
*DUO*: Death Upon Overproduction
E2F: E2 promoter binding Factor
Eo: Eocene
*EXO1*: EXOnuclease 1
*FBP1*: Fructose-1,6-BisPhosphatase 1
FEL: faster-evolving lineage
*GAL*: GALactose metabolism
GC: Guanine–Cytosine
*GDH*: Glutamate DeHydrogenase
gDNA: genomic DNA
*GIP1*: Glycogen 7-Interacting Protein 1
*GLT*: GLuTamate synthase
GO: gene ontology
HMM: Hidden Markov Model
*HSK3*: Helper of ASK1 3
*IMA*: IsoMAltase
*IME1*: Inducer of MEiosis 1
LRT: likelihood ratio test
*MAD1*: Mitotic Arrest-Deficient 1
*MAG1*: 3-MethylAdenine DNA Glycosylase 1
*MAL*: MALtose fermentation
*MALx*: MALtose fermentation locus 1 or 3
MCM: Mini-Chromosome Maintenance
*MDE*: Methylthioribulose-1-phosphate DEhydratase
*MEC3*: Mitosis Entry Checkpoint 3
*MEU*: Multicopy Enhancer of Upstream activation site
MIND: Mis TWelve-like 1 protein Including Necessary for Nuclear Function 1 protein,Nnf1 Synthetic Lethal 1 protein, Dosage Suppressor of Necessary for Nuclear Function 1 protein complex
Mio.: Miocene
*MND2*: Meiotic Nuclear Divisions 2
*MRC1*: Mediator of the Replication Checkpoint 1
*MRI*: MethylthioRibose-1-phosphate Isomerase
*MTW1*: Mis TWelve-like 1
mya: million years ago
*NNF1*: Necessary for Nuclear Function 1
NRRL: Northern Regional Research Laboratory
*NSL1*: Nnf1 Synthetic Lethal 1
OG: orthologous gene
Oligo.: Oligocene
ORC: Origin Recognition complex
Paleo: Paleocene
*PBP2*: Pbp1p Binding Protein 2
*PCD1*: Peroxisomal Coenzyme A Diphosphatase 1
*PCK1*: Phosphoenolpyruvate CarboxyKinase 1
*PCL1*: Pho85 CycLin 1
*PDS1*: Precocious Dissociation of Sisters 1
PHO: PHOsphate metabolism
*PHR1*: PHotoreactivation Repair deficient
Pleisto.: Pleistocene
Plio.: Pliocene
*POL*: POLymerase
Quat: Quaternary
*RAD9*: RADiation sensitive 9
*RFA3*: Replication Factor A
*RFX1*: Regulatory Factor X 1
*RIF1*: Repressor/activator site binding protein-Interacting Factor 1
*SAM*: S-AdenosylMethionine requiring
SBF: Switching deficient 4/6 cell-cycle box-binding factor
SEL: slower-evolving lineage
*SGS1*: Slow Growth Suppressor 1
*SIC1*: Substrate/Subunit Inhibitor of Cyclin-dependent protein kinase 1
*SNO*: SNooze proximal Open reading frame
*SPC*: Spindle Pole Component
*SPE*: SPErmidine auxotroph
*SPO12*: SPOrulation 12
*SSP1*: Sporulation-specific protein 1
*SUC2*: SUCrose 2
*SWM1*: Spore Wall Maturation 1
*TDP1*: Tyrosyl-DNA Phosphodiesterase 1
*THI*: THIamine regulon
UTAD222: University of Trás-os-Montes and Alto Douro 222
*UTR*: Unidentified TRanscript
*WHI5*: WHIskey 5
*YKU70*: Yeast KU protein 70
