# Supplementary figures and images for: Extensive loss of cell-cycle and DNA repair genes in an ancient lineage of bipolar budding yeasts
Source: PLoS Biol. 2019 May 21;17(5):e3000255. doi: 10.1371/journal.pbio.3000255 (PMC6528967; doi:10.1371/journal.pbio.3000255)

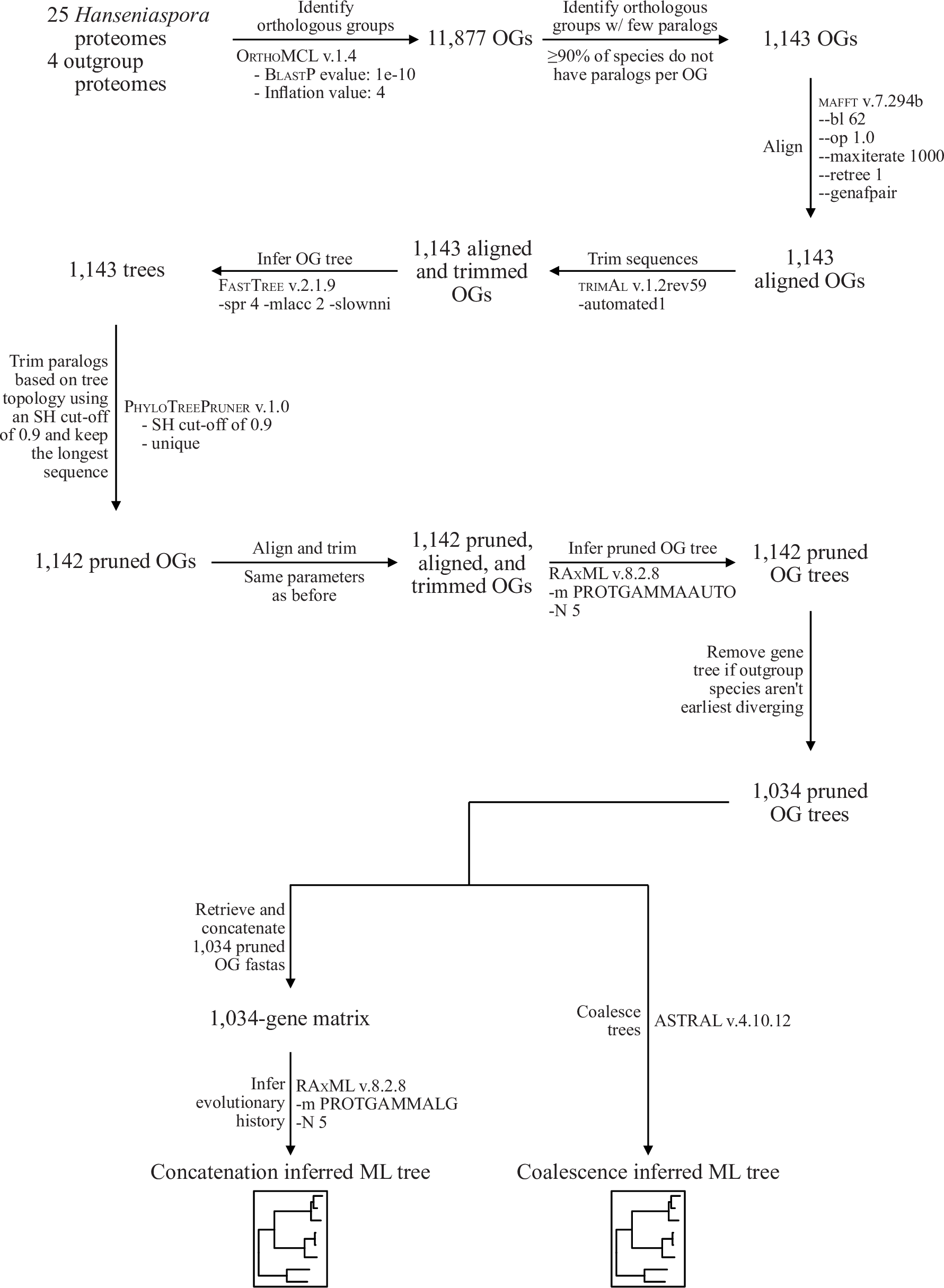

Supplement: S1 Fig — Using 25 Hanseniaspora proteomes and the proteomes of 4 outgroup taxa, we identified 11,877 orthologous groups of genes. For 1,143 orthologous groups, ≥90% of taxa were represented by a single sequence, while the others had two sequences (i.e., putative paralogs). The sequences of the 1,143 orthologous groups were individually aligned, trimmed, had their evolutionary history inferred, and paralogs were trimmed based on tree topology. Using the resulting 1,142 OGs with paralogs trimmed, sequences were realigned, trimmed, and had their evolutionary history inferred. Orthologous groups where the outgroup taxa were not recovered as the sister clade to the genus Hanseniaspora were removed, reducing the set to 1,034 orthologous groups. Among these 1,034 orthologous groups of genes, a concatenated 1,034-gene matrix was constructed and used for reconstructing evolutionary history. Similarly, evolutionary history was inferred using coalescence of the 1,034 orthologous group single-gene phylogenies. OG, orthologous gene. (TIF) [file pbio.3000255.s001.tif]

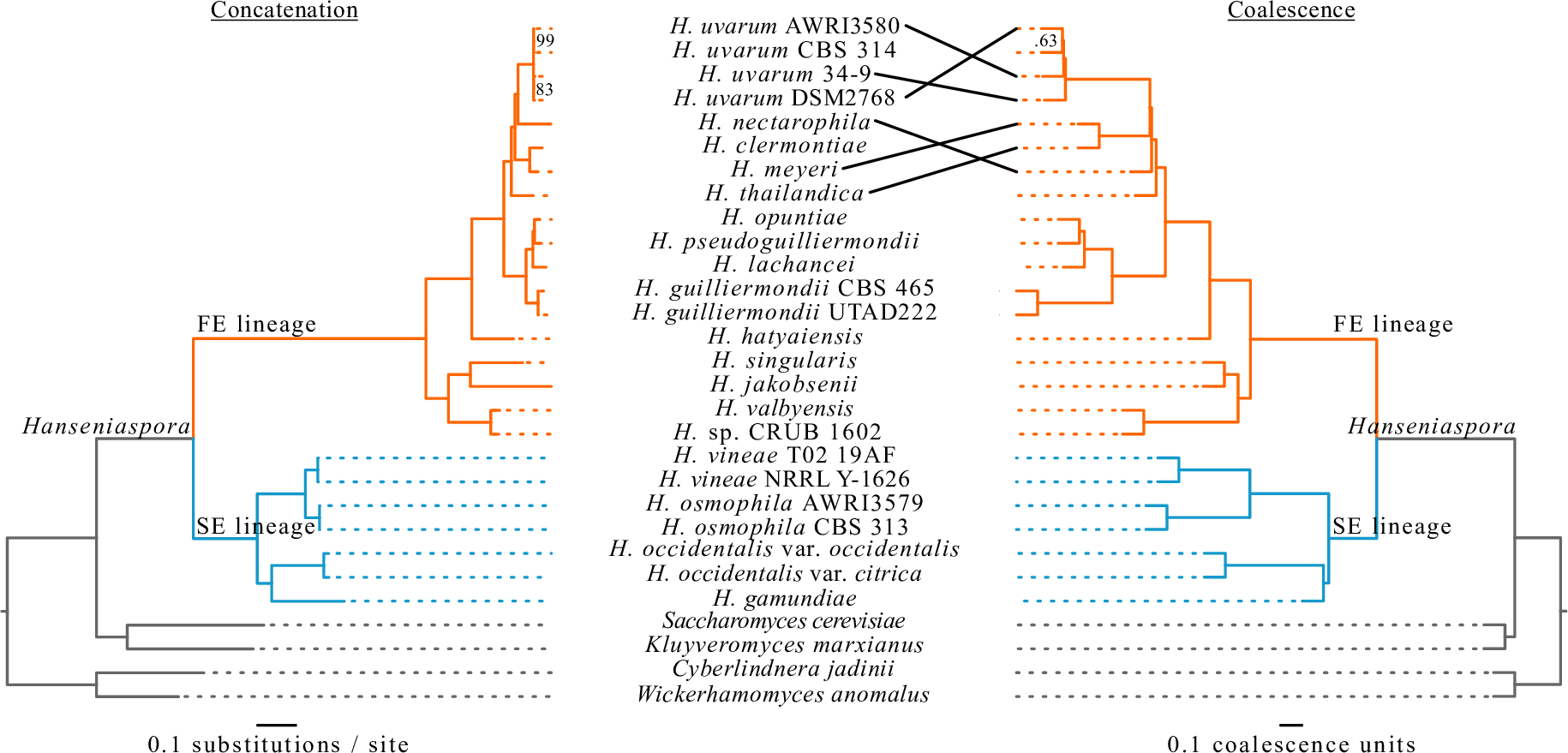

Supplement: S2 Fig — (Left) Concatenation provides support for a lineage with a long stem branch, which we term the FEL, and another lineage with a much shorter stem branch, which we term the SEL. (Right) Coalescence supports monophyly of the FEL and SEL. Minor discrepancies are observed between the topologies. Only the values for bipartitions without full support are shown. Support for concatenation and coalescence was determined using 100 rapid bootstrap replicates and local posterior support, respectively. figshare: https://doi.org/10.6084/m9.figshare.7670756.v2. FEL, faster-evolving lineage; SEL, slower-evolving lineage. (TIF) [file pbio.3000255.s002.tif]

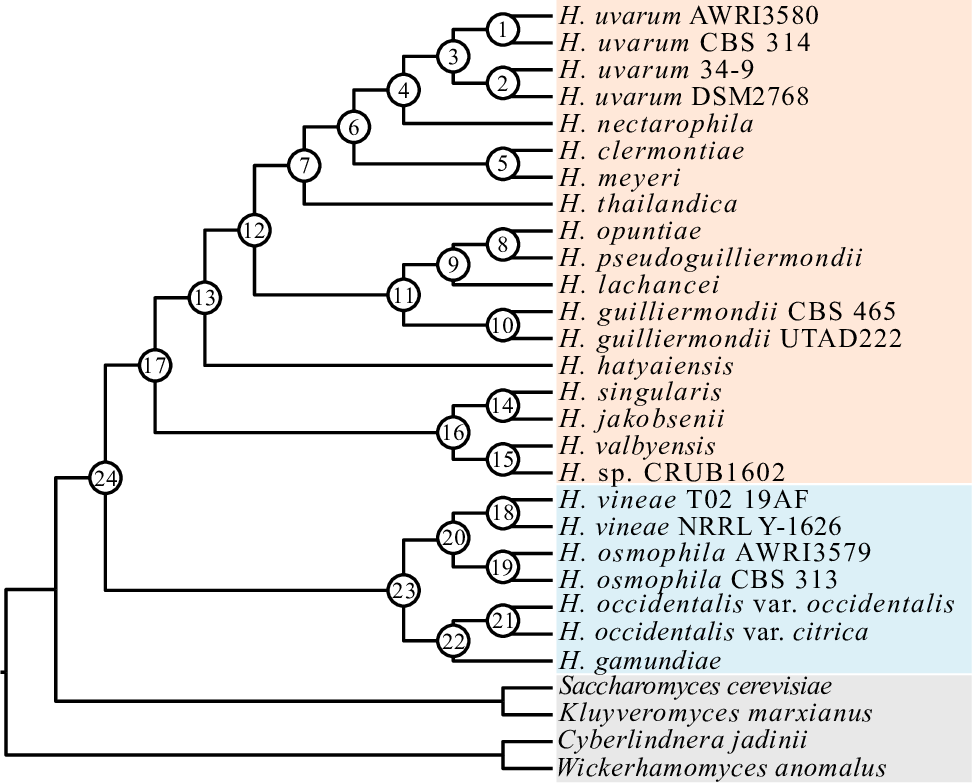

Supplement: S3 Fig — Internode identifiers for time tree analysis are shown in Fig 1B. Associated mean divergence time and credible intervals can be found in the S2 File. (TIF) [file pbio.3000255.s003.tif]

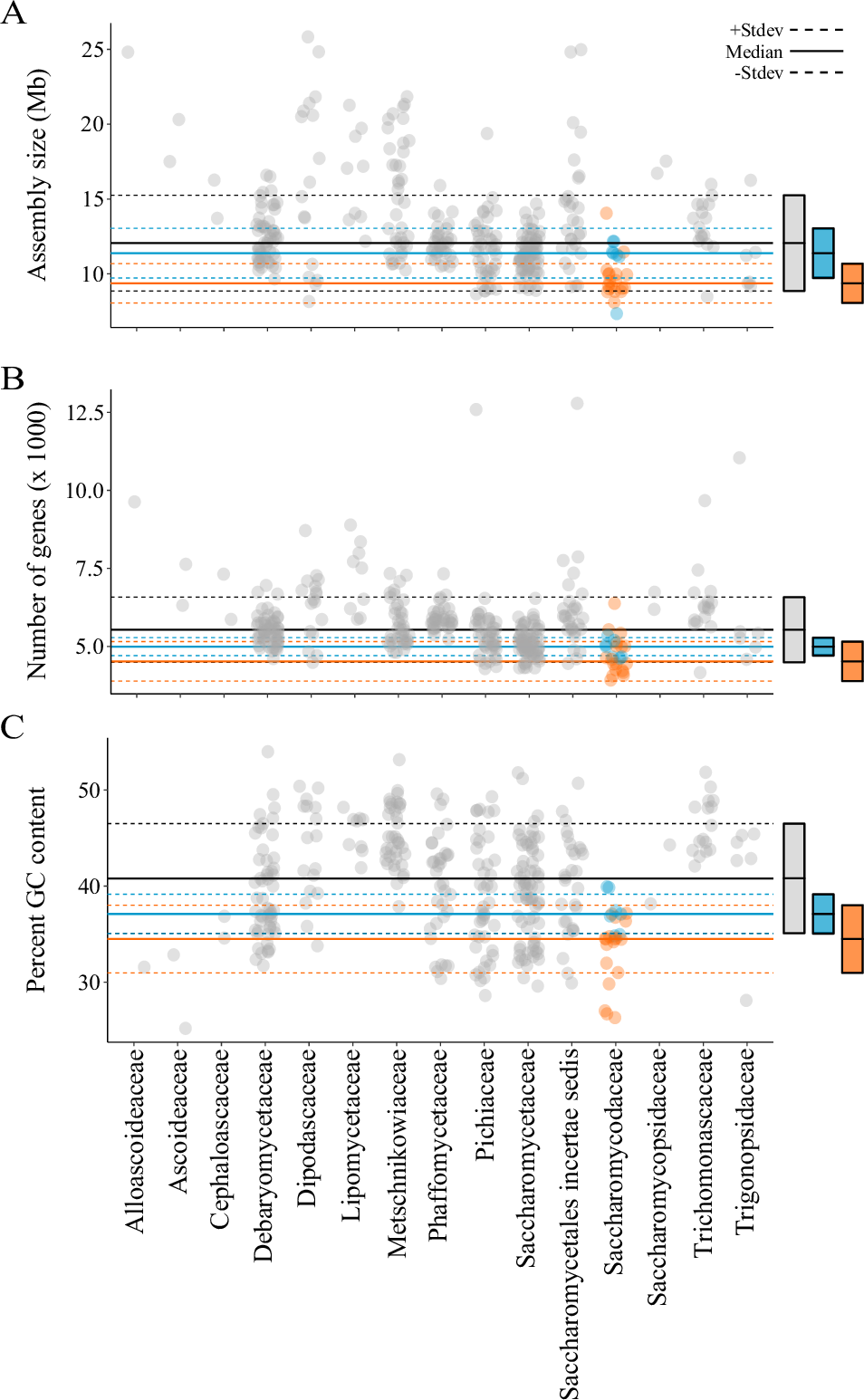

Supplement: S4 Fig — (A) The genus Hanseniaspora (family Saccharomycodaceae) includes the smallest budding yeast genome. Average genome sizes in the FEL, SEL, and the Saccharomycotina are 9.71 ± 1.32 Mb (min: 8.10; max: 14.05), 10.99 ± 1.66 Mb (min: 7.34; max: 12.17), 12.80 ± 3.20 Mb (min: 7.34; max: 25.83), respectively. (B) The genus Hanseniaspora includes the budding yeast genome with the fewest genes. Average number of genes per genome in the FEL, SEL, and Saccharomycotina are 4,707.89 ± 633.56 (min: 3,923; max: 6,380), 4,932.43 ± 289.71 (min: 4,624; max: 5,349), and 5,657.66 ± 1,044.78 (min: 3,923; max: 12,786), respectively. (C) The genus Hanseniaspora has among the lowest GC content values in budding yeast genomes. GC content values in the FEL, SEL, and Saccharomycotina are 33.10 ± 3.53% (min: 26.32; max: 37.17), 37.28 ± 2.05% (min: 34.82; max: 39.93), and 40.30 ± 5.71% (min: 25.2; max: 53.98), respectively. Families of Saccharomycotina are depicted on the y-axis. Median values are depicted with a line, and dashed lines indicate plus or minus one standard deviation from the median. To the right of each figure, boxplots depict the median and standard deviations of each grouping. The gray represents all of Saccharomycotina. Blue represents the SEL, and orange represents the FEL. figshare: https://doi.org/10.6084/m9.figshare.7670756.v2. FEL, faster-evolving lineage; GC, Guanine–Cytosine; max, maximum; min, minimum; SEL, slower-evolving lineage. (TIF) [file pbio.3000255.s004.tif]

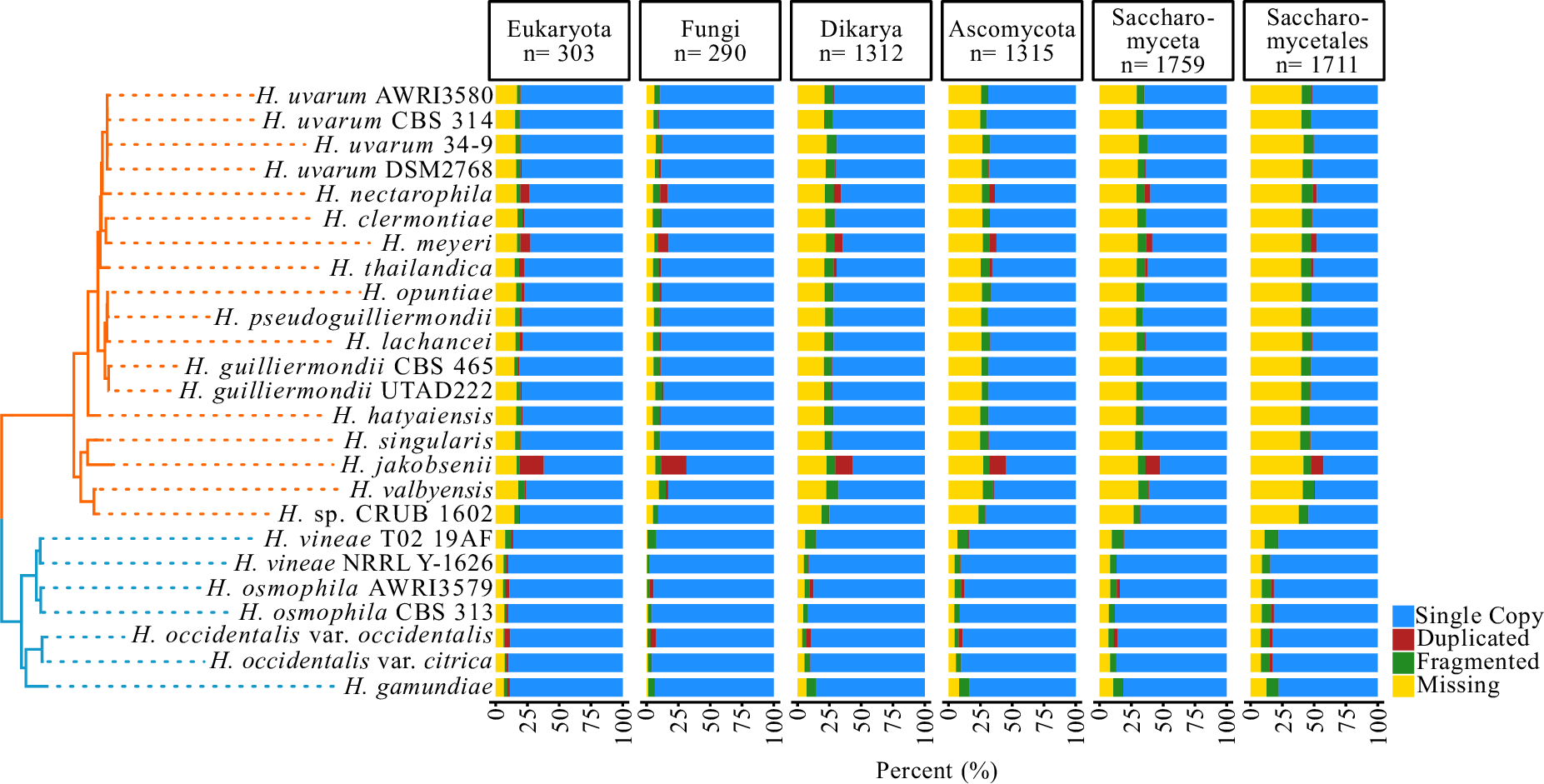

Supplement: S5 Fig — BUSCO [120] analyses of Hanseniaspora proteomes using the Eukaryota (nBUSCOs = 303), Fungi (nBUSCOs = 290), Dikarya (nBUSCOs = 1,312), Ascomycota (nBUSCOs = 1,315), Saccharomyceta (nBUSCOs = 1,759), and Saccharomycetales (nBUSCOs = 1,711) orthoDB databases revealed that very large numbers of BUSCO genes are absent from Hanseniaspora genomes and from FEL genomes in particular. figshare: https://doi.org/10.6084/m9.figshare.7670756.v2. BUSCO, Benchmarking Universal Single-Copy Orthologs; FEL, faster-evolving lineage. (TIF) [file pbio.3000255.s005.tif]

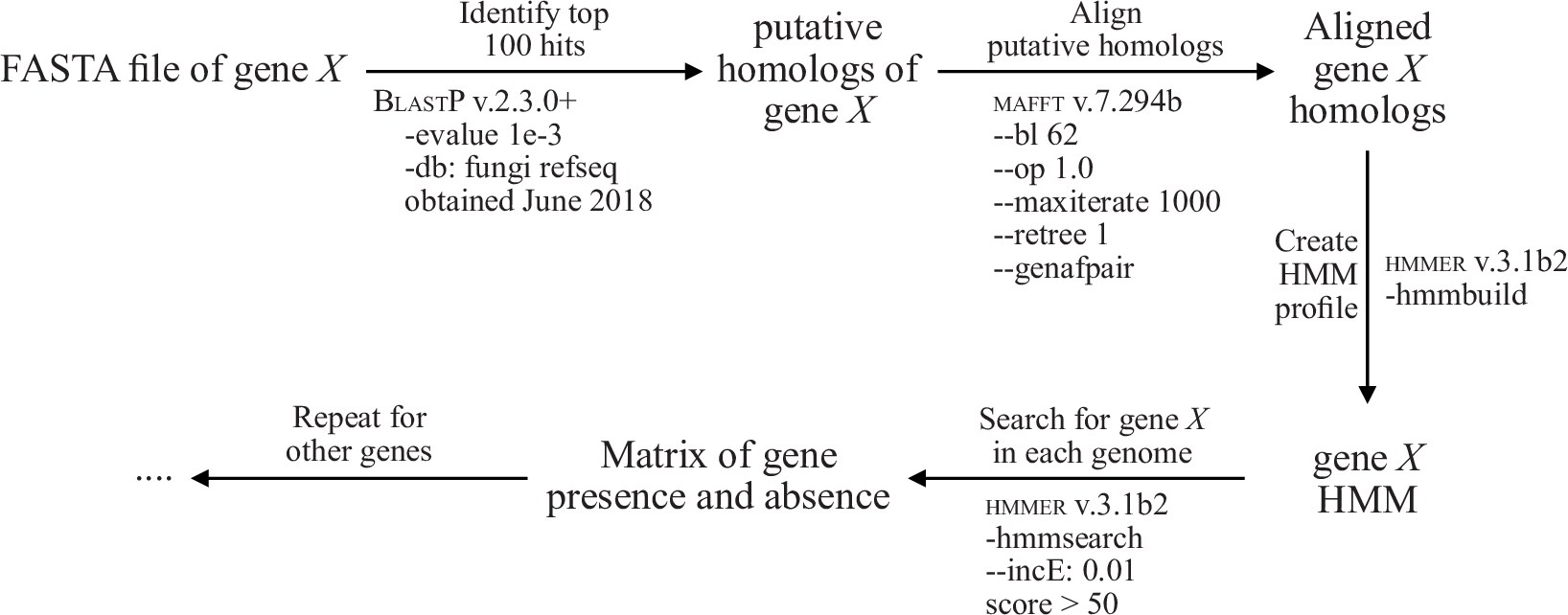

Supplement: S6 Fig — (A) A FASTA file for gene X, where gene X is the FASTA entry of a verified ORF in the S. cerevisiae proteome, was used as a query to search for putative homologs in the Fungal reference sequence (refseq) database. The top 100 putative homologs were subsequently aligned. From the alignment, an HMM was made. Using the HMM, gene X was searched for in the genome of each species from the FEL, SEL, and outgroup individually using a liberal e-value cutoff of 0.01 and a score of >50. This pipeline yields presence and absence information of gene X among FEL, SEL, and outgroup taxa. This method was subsequently applied to all verified ORFs in the S. cerevisiae proteome. FEL, faster-evolving lineage; HMM, Hidden Markov Model; ORF, open reading frame; refseq, reference sequence; SEL, slower-evolving lineage. (TIF) [file pbio.3000255.s006.tif]

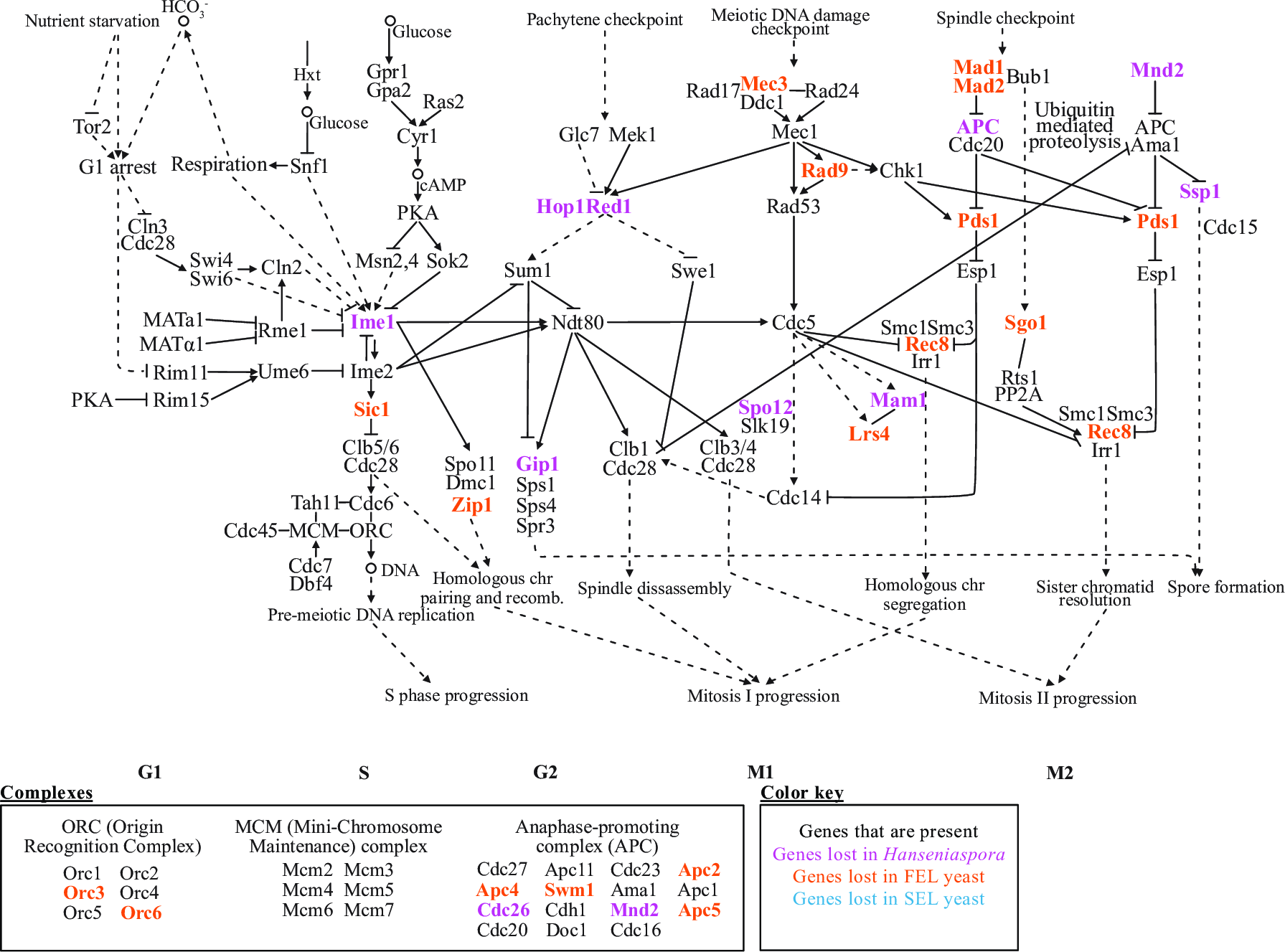

Supplement: S7 Fig — Gene presence and absence analysis of genes that participate in the gluconeogenesis (A) and glycolysis (B) pathway reveal the absence of key genes in the gluconeogenesis pathway, suggestive of a diminished capacity for gluconeogenesis. More specifically, PCK1, which encodes the enzyme that converts oxaloacetic acid to phosphoenolpyruvate, and FBP1, which encodes the enzyme that converts fructose-1,6-bisphosphate to fructose-6-phospbate, are absent from all Hanseniaspora species. figshare: https://doi.org/10.6084/m9.figshare.7670756.v2. FBP1, Fructose-1,6-BisPhosphatase 1; PCK1, Phosphoenolpyruvate CarboxyKinase 1. (TIF) [file pbio.3000255.s007.tif]

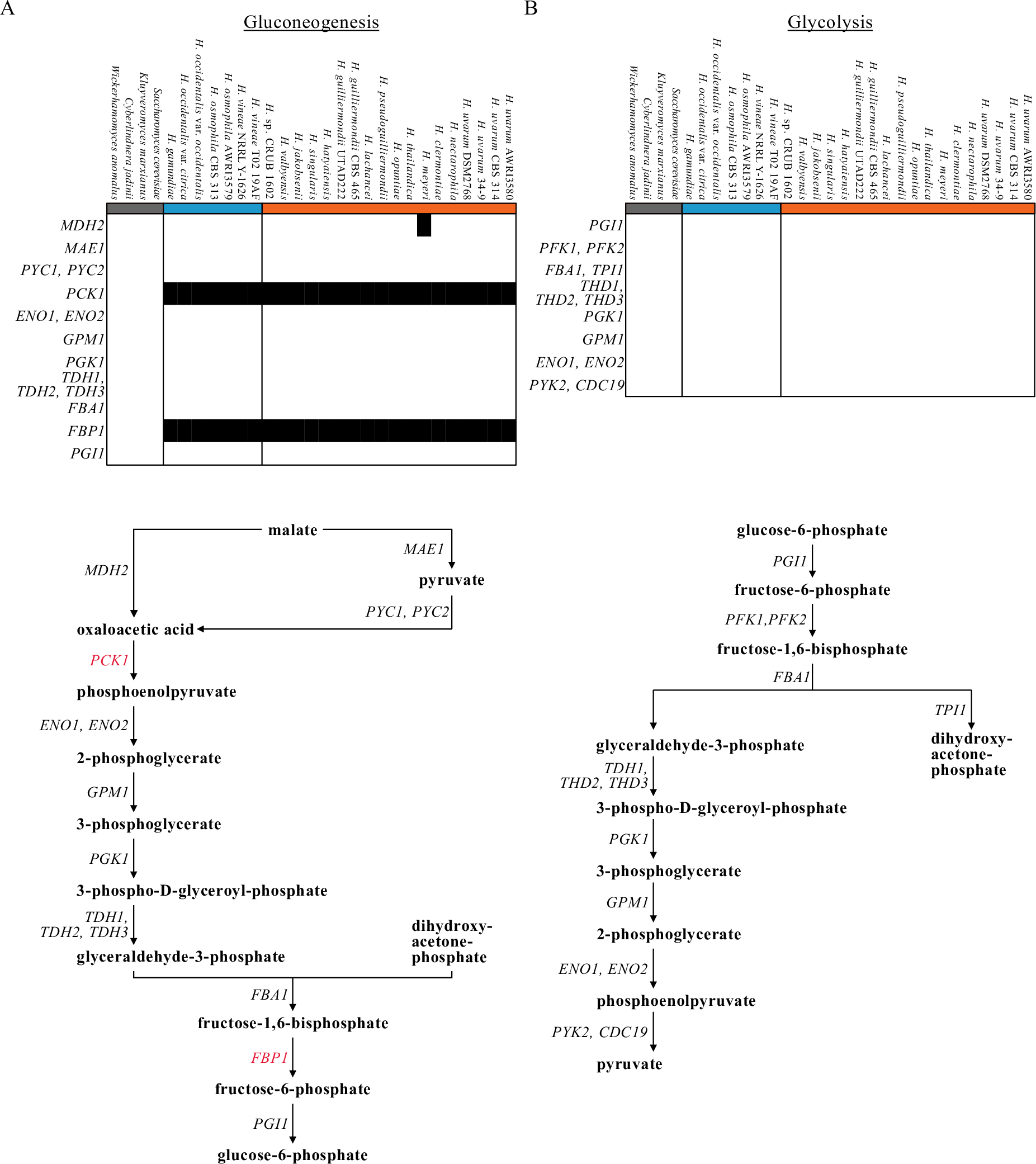

Supplement: S8 Fig — (A) Plots with peaks at high and low base frequencies (or a “smiley pattern”) reflect a lack of biallelic sites, which is suggestive of a haploid genome. The smiley-pattern distributions observed for the genomes of H. occidentalis var. occidentalis, H. uvarum CBS 314, and H. guilliermondii CBS 465 suggest these species have haploid genomes. (B) A unimodal distribution centered around a base frequency of 50 is consistent with the presence of two alleles at a given locus and suggestive of a diploid genome. The unimodal distributions centered around a base frequency of 50 suggest H. occidentalis var. citrica, H. osmophila CBS 313, H. meyeri, H. clermontiae, H. nectarophila, H. thailandica, H. pseudoguilliermondii, H. singularis, and K. hatyaiensis are diploids. (C) Bimodal distributions centered around base frequencies of 33 and 66 reflect one allele on one chromosome and another allele on the other two chromosomes, which is suggestive of a triploid genome. Bimodal distributions centered around 33 and 66 suggest H. lachancei and H. jakobsenii are triploid. (D) Analyses of H. vineae CBS 2171, H. valbyensis, H. sp. NRRL Y-63759, and H. opuntiae base frequency distributions were ambiguous. Certain FEL species, such as H. singularis, H. pseudoguilliermondii, and H. jakobsenii, are potentially aneuploid, while evidence of aneuploidy in the SEL is observed only in H. occidentalis var. citrica. figshare: https://doi.org/10.6084/m9.figshare.7670756.v2. CBS, Centraalbureau voor Schimmelcultures; FEL, faster-evolving lineage; NRRL, Northern Regional Research Laboratory; SEL, slower-evolving lineage. (TIF) [file pbio.3000255.s008.tif]

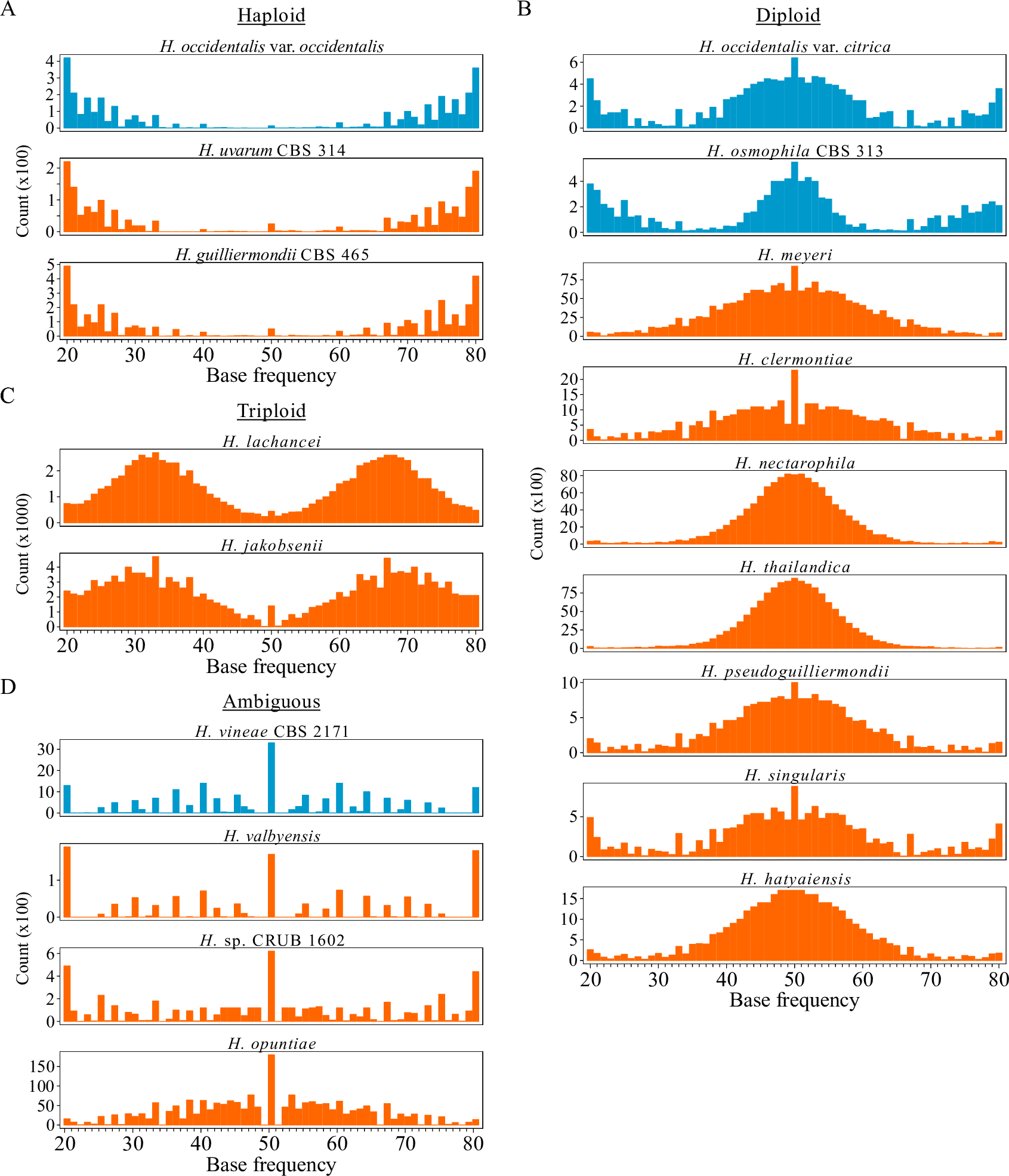

Supplement: S9 Fig — Genes absent in both lineages and the FEL are colored purple and orange, respectively. Dotted lines with arrows indicate indirect links or unknown reactions. Lines with arrows indicate molecular interactions or relations. Circles indicate chemical compounds such as glucose or cAMP. figshare: https://doi.org/10.6084/m9.figshare.7670756.v2. cAMP, cyclic AdenosineMonoPhosphate; FEL, faster-evolving lineage. (TIF) [file pbio.3000255.s009.tif]

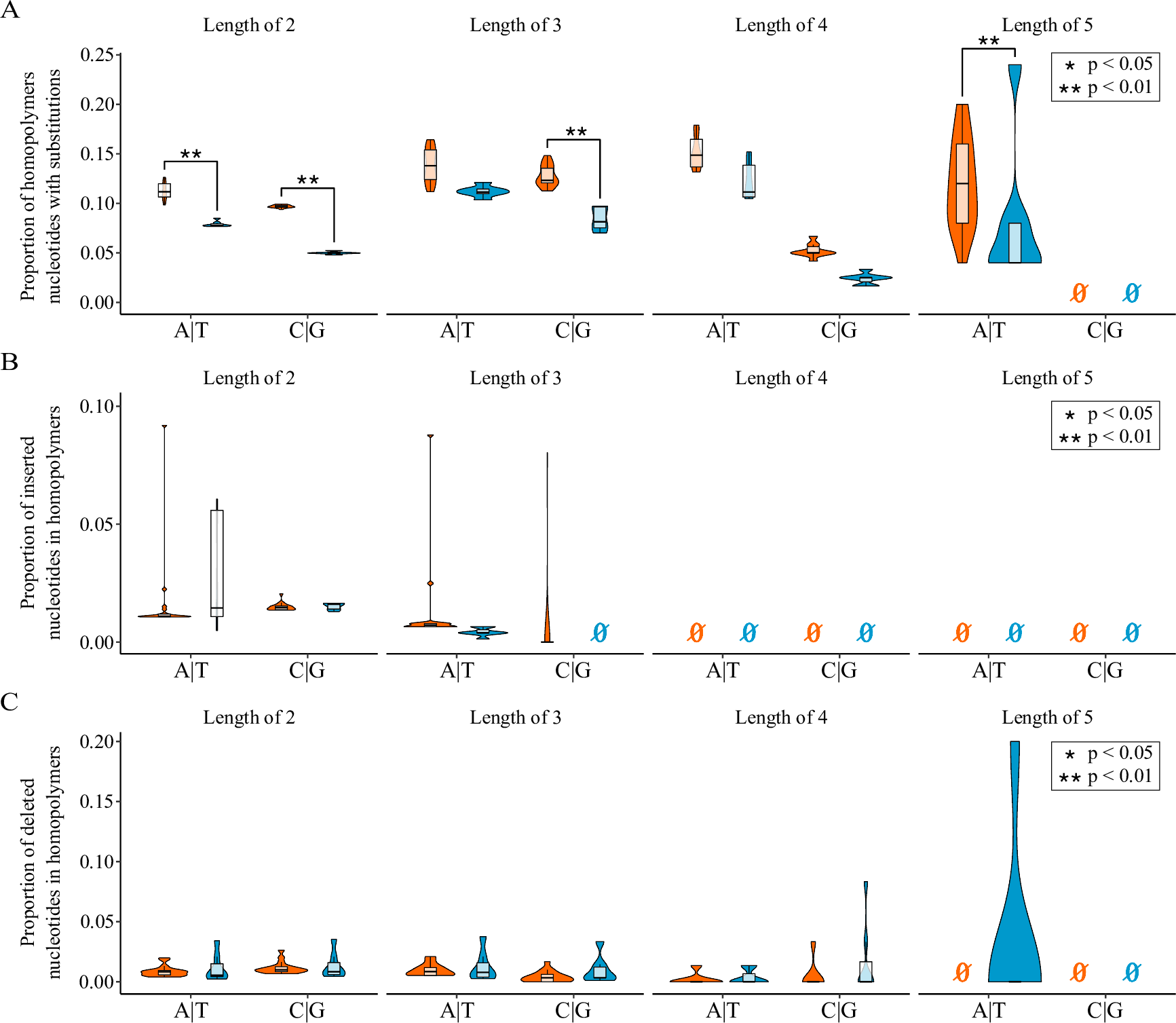

Supplement: S10 Fig — Significant differences among the proportion of mutated bases among homopolymers of various lengths were observed (Fig 6). Addition of variables (i.e., sequence type [A|T or C|G] and mutation type [base substitution, insertion, and deletion]) allowed for further determination of what types of mutations caused differences between the FEL and SEL. As shown in Fig 6, we observed significant differences in the numbers of mutations between the FEL and SEL (F = 27.06, p < 0.001; multifactor ANOVA) as well as in the type of mutations (F = 1686.70, p < 0.001; multifactor ANOVA). A Tukey honest significance differences post hoc test revealed that the proportion of nucleotides that underwent base substitutions was significantly greater than insertions (p < 0.001) and deletions (p < 0.001). We next focused on significant differences observed between the FEL and SEL when considering all factors. We observed significant differences between the FEL and SEL at A|T and C|G homopolymers with a length of 2 (p = 0.009 and p < 0.001, respectively), C|G homopolymers of length 3 (p < 0.001), and A|T homopolymers of length 5 (p < 0.001). figshare: https://doi.org/10.6084/m9.figshare.7670756.v2. FEL, faster-evolving lineage; SEL, slower-evolving lineage. (TIF) [file pbio.3000255.s010.tif]

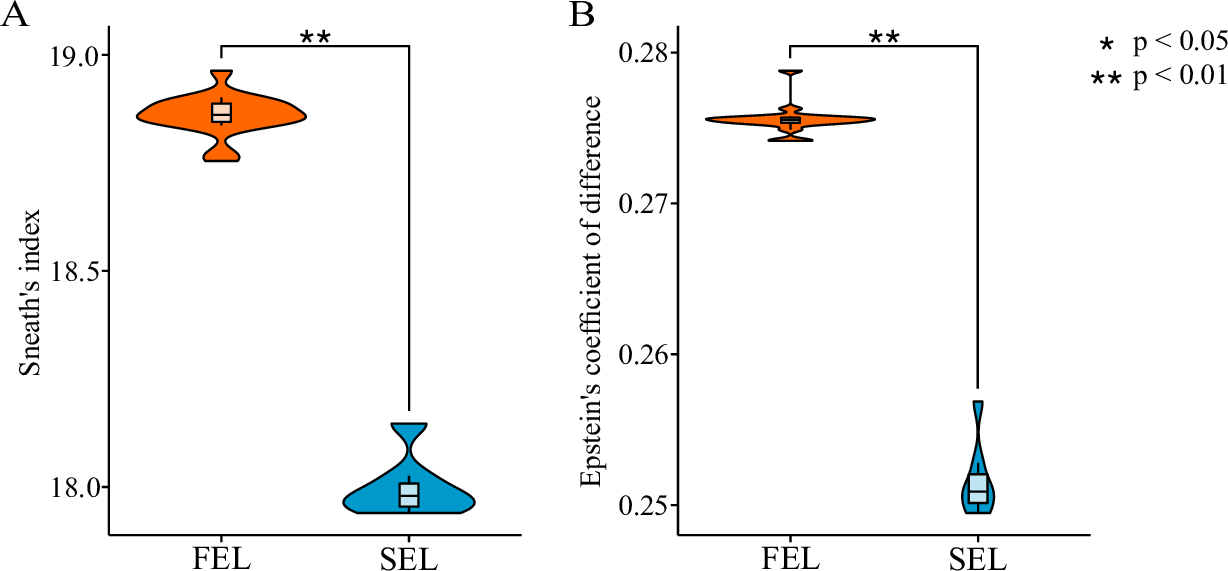

Supplement: S11 Fig — Using Sneath’s index and Epstein’s coefficient of difference, the average difference among amino acid substitutions were determined among sites where the outgroup taxa had all the same amino acid. Using either metric, amino-acid substitutions were significantly more radical in the FEL compared to the SEL (p < 0.001; Wilcoxon rank–sum test for both metrics). figshare: https://doi.org/10.6084/m9.figshare.7670756.v2. FEL, faster-evolving lineage; SEL, slower-evolving lineage. (TIF) [file pbio.3000255.s011.tif]

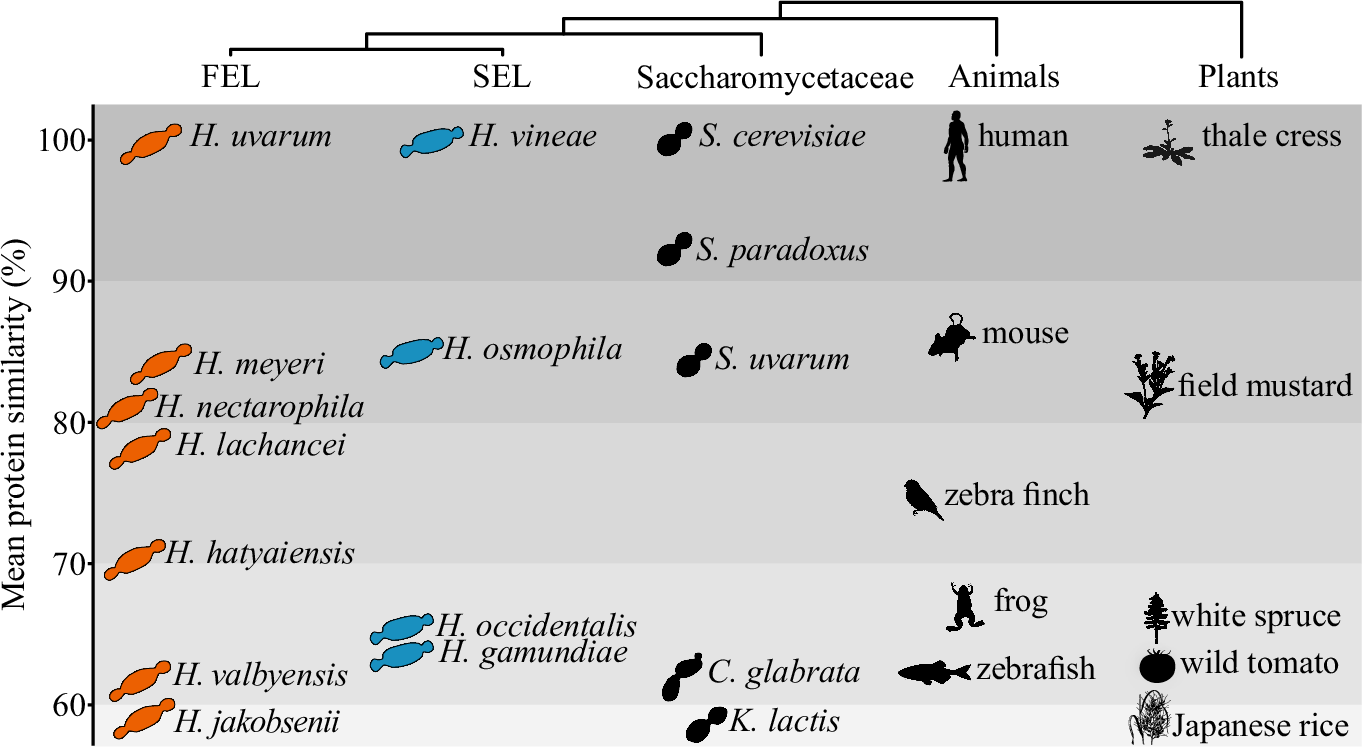

Supplement: S12 Fig — Mean protein similarity (measured by amino acid substitutions/site) between species in the FEL (using H. uvarum as the reference), species in the SEL (using H. vineae as a reference), Saccharomycetaceae (S. cerevisiae), animals (human), and plants (thale cress Arabidopsis thaliana). For each lineage, mean protein similarity was estimated using a reciprocal best blast hit approach. The mean protein similarity observed in these lineages is roughly on par with genus-level differences within the family Saccharomycetaceae, humans to zebrafish, and thale cress to Japanese rice. Silhouettes were obtained under the Public Domain or Creative Commons license from phylopic.org (human, mouse, zebra finch, frog, zebrafish, and thale cress), from openclipart.org (field mustard, white spruce, wild tomato, Japanese rice; the colors of the field mustard and wild tomato original images were changed to black), or drawn by hand by Jacob L. Steenwyk (all yeasts). FEL, faster-evolving lineage; SEL, slower-evolving lineage. (TIF) [file pbio.3000255.s012.tif]
